# Navigating the risks: a systematic review of immune checkpoint inhibitor therapy before liver transplant for hepatocellular carcinoma and its impact on allograft rejection and survival outcomes

**DOI:** 10.3389/fonc.2025.1689820

**Published:** 2025-10-29

**Authors:** Donghua Liu, Xinyi Wang, Xuelian Liu, Jing Li

**Affiliations:** Department of Pharmacy, The Affiliated Hospital of Qingdao University, Qingdao, Shandong, China

**Keywords:** hepatocellular carcinoma, immune checkpoint inhibitors, liver transplant, allograft rejection, meta-analysis

## Abstract

**Background:**

The administration of immune checkpoint inhibitors (ICIs) prior to liver transplantation (LT) for hepatocellular carcinoma (HCC) has been reported. Several studies suggest that ICIs may elevate the risk of allograft rejection (AR) and influence other clinical outcomes. This meta-analysis aimed to assess the efficacy and safety of pre-LT ICI treatment in HCC patients.

**Methods:**

A systematic literature search was conducted in PubMed, Embase, Cochrane, and Web of Science for retrospective studies and randomized controlled trials (RCTs) examining pre-LT ICI therapies in HCC patients. Random-effects models were employed to evaluate treatment effects on allograft rejection (AR), complete recovery rate among patients with AR, graft loss, HCC recurrence, and progression-free survival (PFS). Common-effects models were used to assess overall mortality and AR-related mortality. Study quality was evaluated using the JBI critical appraisal tools. The review was registered with PROSPERO (CRD42024616267).

**Results:**

Studies involving HCC patients receiving pre-LT ICIs for downstaging or bridging were included. After screening databases from inception to 31 December 2024, eight studies (*n* = 229 patients) were included. The studies had diverse designs and were primarily from China and the US. The pooled post-LT AR rate across all eight studies was 19% (95% CI: 12%–30%). The incidence of AR was 24% in the PD-L1 inhibitor group, 18% in the PD-1 inhibitor group, and 20% in the bispecific/combination therapies group. The complete recovery rate among patients with AR was 78% (95% CI: 59%–97%), and graft loss occurred in 4% (95% CI: 1%–7%). The HCC recurrence rate across six studies was 24% (95% CI: 12%–36%). The pooled median recurrence-free survival (RFS) was 17.63 months (95% CI: 11.57–23.69 months). Overall mortality was 8% (95% CI: 4%–12%), and AR-related mortality was 2% (95% CI: 0%–5%). Sensitivity analysis supported the robustness of the results, while funnel plots indicated potential publication bias for several outcomes. This meta-analysis offers a comprehensive synthesis of the impact of pre-LT ICIs on post-transplantation outcomes.

**Conclusion:**

The use of ICIs as bridging or downstaging therapy prior to liver transplantation in HCC patients appears feasible.

**Systematic review registration:**

https://www.crd.york.ac.uk/prospero/, identifier CRD42024616267.

## Introduction

According to global cancer statistics 2022, liver cancer ranks as the sixth most commonly diagnosed cancer and the third leading cause of cancer-related deaths globally (1). Notably, hepatocellular carcinoma (HCC) accounts for 75%–85% of primary liver cancer cases (2). The pathogenesis of HCC is tightly linked to chronic liver injury and inflammation. Hepatitis B virus (HBV) and hepatitis C virus (HCV) infections are major risk factors for the development of HCC. In addition, exposure to aflatoxin, heavy alcohol consumption, obesity, type 2 diabetes, and smoking are also associated with an increased risk of HCC (2). Clinically, HCC is characterized by “silent progression” in its early stages. Owing to the liver’s robust regenerative capacity, early-stage HCC patients typically lack specific symptoms, with only mild, non-specific complaints (e.g., fatigue, anorexia) in rare cases. This leads to delayed diagnosis: approximately 60%–70% of patients are diagnosed at intermediate or advanced stages, when curative treatment options are limited. Consequently, the overall prognosis of HCC remains poor, with a 5-year survival rate of less than 10% for advanced-stage patients—underscoring the urgent need for improved screening tools and therapeutic strategies (3).

In the current clinical management paradigm, treatment strategies for HCC are stratified by disease stage and liver function. For early-stage HCC patients with well-preserved liver function (Child–Pugh Class A), surgical resection is the first-line curative option. However, for early-stage patients with decompensated liver function (e.g., Child–Pugh Class B/C) or those with small tumors but poor hepatic reserve (e.g., cirrhotic liver with portal hypertension), liver transplantation (LT) is the preferred treatment. LT offers a dual benefit: it removes the tumor and replaces the diseased liver, achieving long-term disease control in eligible patients. Additionally, LT is applicable to patients with locally advanced HCC whose viable tumor burden is reduced to within acceptable transplant criteria [e.g., Milan criteria, University of California San Francisco (UCSF) criteria] following locoregional therapies (LRTs) or systemic therapy (4, 5).

Immunotherapy based on immune checkpoint inhibitors (ICIs) has made significant progress in the treatment of advanced HCC. Currently, the approved ICIs mainly include three categories: programmed cell death protein 1 (PD-1) inhibitors, programmed cell death 1 ligand 1 (PD-L1) inhibitors, and cytotoxic T lymphocyte-associated protein 4 (CTLA-4) inhibitors. The National Comprehensive Cancer Network (NCCN) recommends ICIs as a first-line or subsequent-line treatment for advanced HCC (6). In multiple phase 3 clinical studies, ICIs in combination with other compounds have demonstrated meaningful improvements in patients with advanced HCC (7, 8). The IMbrave050 trial demonstrated that atezolizumab plus bevacizumab yielded significantly higher overall survival (OS) and progression-free survival (PFS) than sorafenib alone in patients with advanced or unresectable HCC (7). Currently, several new trials are being carried out to explore the use of ICIs to replace or supplement LRTs in treating patients with unresectable HCC. A recent randomized clinical trial (RCT) reported that, in unresectable HCC amenable to embolization, the combination of durvalumab, bevacizumab, and transarterial chemoembolization (TACE) outperforms TACE alone in terms of objective responses and PFS (9). Small-scale trials have also shown that ICI treatment prior to resection has promising efficacy for patients with resectable HCC (10).

Although the indications for ICI treatment are expected to expand to a broader population of HCC patients, the use of ICI prior to LT still presents potential concerns regarding the increased risk of allograft rejection (AR) (11). The period (the time from the last ICI treatment to LT) and the type of ICIs may be related to AR risk (11, 12). In recent years, an increasing number of retrospective studies and RCTs have been attempting to demonstrate that ICIs are a potential treatment strategy for bridging or downstaging prior to LT (13–16). The PLENTY pilot study is the first RCT to assess the efficacy of ICIs in LT recipients diagnosed with HCC beyond the Milan criteria (MC). This study demonstrated that pembrolizumab plus lenvatinib yielded a favorable objective response and improved recurrence-free survival (RFS) without increasing AR after LT (16). However, a notable limitation of this study is its relatively small sample size. Given the limited sample size of reports, the safety data on the use of ICIs prior to LT remain inadequate. Consequently, we conducted a meta-analysis of relevant retrospective trials and RCTs to determine the impact of using ICIs before LT on post-transplantation outcomes, including AR, HCC recurrence, and mortality.

## Methods

This review was conducted and reported according to the Preferred Reporting Items for Systematic Reviews and Meta-Analyses (PRISMA) guidelines (17). The protocol of our study was registered in the International Prospective Register of Systematic Reviews (PROSPERO)—CRD42024616267.

### Inclusion and exclusion criteria

The inclusion criteria of included studies were as follows: 1) population: patients diagnosed with HCC; 2) intervention: recipients who had undergone pre-LT ICI therapy for downstaging or bridging during their waiting period for LT; 3) study type: retrospective studies and RCTs; 4) outcomes: the clinical outcomes of interest were reported, including AR, the full recovery rate of patients with AR, graft loss, PFS, HCC recurrence, overall mortality, and AR-related mortality; and 5) all studies must be written in English or Chinese. The exclusion criteria were as follows: 1) articles that only mentioned methods or protocols; and 2) reviews, guidelines, case reports, and conference abstracts.

### Database search strategy and screening process

Two independent investigators retrieved relevant studies from PubMed, Embase, Cochrane, and Web of Science. The search period was from database inception to 31 December 2024. The search terms (HCC, LT, and ICIs) were based on three key concepts and adapted for each database search. The full search formulas for each database are provided in the **Supplementary Appendix**.

After the initial literature search was completed, duplicate reports were removed upon identification using EndNote. Two investigators excluded irrelevant records by screening titles and abstracts and further excluded studies that did not meet the inclusion criteria by reading the full text. In the studies of overlapping patient cases, we included the most recent study or the publication with the most complete data. In the event of a disagreement between the investigators, the final determination was made in collaboration with the senior investigator.

### Data extraction and risk of bias assessment

Two investigators independently extracted the key characteristics and outcomes from the eligible studies, as well as from the relevant **Supplementary Materials**, by strictly following a predefined data extraction table. The data extracted included authors, year of publication, country of origin, study design, sample size, patient age, treatment regimens, follow-up, and reported outcomes (AR, HCC recurrence, RFS, mortality, etc.). PFS was defined as the time elapsed until radiological evidence of tumor recurrence after LT. Data extraction was meticulously performed by one investigator and subsequently cross-checked for accuracy by another investigator.

The risk of bias was evaluated by two seasoned investigators to ensure objectivity and reliability of the assessment process. All studies were assessed using the JBI critical appraisal tools (18). For each domain, a determination was made and assigned as either “yes,” “no,” “unclear,” or “not applicable.” Any discrepancies regarding the quality assessment were amicably resolved upon confirmation by a senior investigator.

### Statistical analysis

The R software (version 4.1.2) was used for data analysis (http://www.r-project.org/). Heterogeneity was evaluated using the chi-squared test along with the *I*² statistic. A *p*-value ≤0.05 was indicative of a statistically significant difference. If significant heterogeneity was present, as determined by a *p*-value ≤0.05 and I^2^ >50%, a random-effects model was then applied for the analysis. Otherwise, the common-effects model was used. Moreover, a sensitivity analysis was meticulously conducted to evaluate the stability and reliability of the results. Finally, funnel plots were used to assess the publication bias.

## Results

### Search results

**Figure 1:**
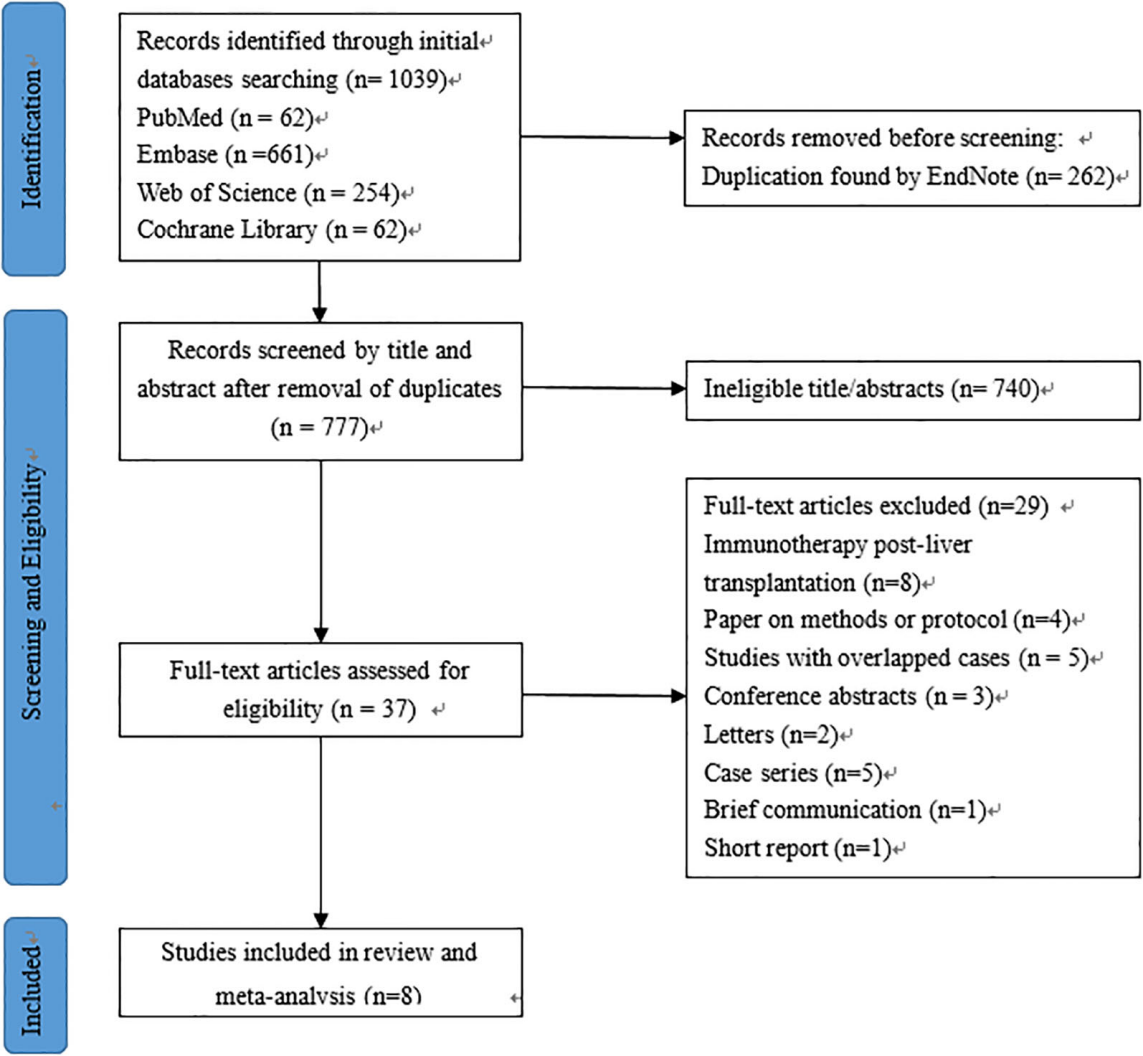
PRISMA flowchart of article selection.

The initial search yielded a total of 1,039 published relevant studies from four databases (PubMed = 62, Embase = 661, Web of Science = 254, and Cochrane Library = 62). Approximately 37 studies were retained after removing duplicates and screening the titles and abstracts. The remaining full-text articles were carefully assessed, and eight studies (*n* = 229 patients) were included in the meta-analysis (13–16, 19–22). The number of studies included at each stage of the selection process was outlined in the PRISMA flow diagram (**Figure 1**).

### Characteristics of the included studies

**Table 1** describes the characteristics of all studies included in the systematic review. Seven studies (13, 14, 16, 19–22) were conducted in China, and one study (15) was conducted in the United States between 2021 and 2024.

**Table 1:** Characteristics of the included studies.

Study (year published)	Country	Study types	Patients	Sample sizes (M/F)	Donor graft type (*n*)	Mean age (years)	ICIs (*n*)	Reasons for ICI therapy.	ICI cycles	Period (days/months)	Follow-up time (days/months)
Xu et al. (2024) (13)	China	Multicenter retrospective study	Beyond LT criteria	25 (21/4)	DD (23) LD (2)	54 (48–57.5)	Camrelizumab (6) Sintilimab (4) Pembrolizumab (4) Nivolumab (1) Toripalimab (1) Penpulimab (1) Envafolimab (1) Cadonilimab (1) Sequential therapy (6)	Downstaging	6 (2–10)	64 (40–150.75) days	27 (18–35) months
Guo et al. (2024) (14)	China	Multicenter retrospective study	Within or beyond LT criteria	83 (72/11)	DBD (62) DCD (21)	>50, 32.5%	Camrelizumab (31) Pembrolizumab (18) Sintilimab (14) Tislelizumab (11) Nivolumab (4) Atezolizumab (5)	Bridging(15) Downstaging (68)	4.0 (2.0–6.0)	58.0 (29.0–110.0) days	8.1 (3.3–14.6) months
Tabrizian et al. ^(^2024^)^ (15)	USA	Prospective study	Within or beyond LT criteria	43, NR	DD (40) LD (3)	NR	Nivolumab Atezolizumab/bevacizumab Pembrolizumab Durvalumab/tremelimumab	Bridging or downstaging	7.5 (4–13.5)	43 (13–120) days	NR
Lv et al. (2024) (16)	China	RCT	Beyond MC and without extrahepatic spread	10, 9/1	DCD	57.5 (38–68)	Pembrolizumab (10)	Downstaging	4 (range 2–5)	60.5 (range 25–193) days	33.4 (range 23.1–45.0) months
Lu et al. (2024) (19)	China	Retrospective cohort study	Within or beyond LT criteria	39 (36/3)	NR	51 (36–71)	Tislelizumab (11) Sintilimab (10) Camrelizumab (7) Pembrolizumab (6) Toripalimab (2) Durvalumab (2) Atezolizumab (1)	Bridging or downstaging	4 (range 1–24)	50 (range 3–840) days	NR
Wang et al. (2023) (20)	China	Retrospective cohort study	Beyond LT criteria	16, 14/2	DBD	50.5 (46.75–55)	Nivolumab (2) Pembrolizumab (7) Sintilimab (4) Camrelizumab (2) Nivolumab, toripalimab, sintilimab, and tislelizumab (1)	Downstaging	4 (range 1–27)	28.5 (range 7–184) days	352.5 (325.2–758.8) days
Duan et al. (2022) (21)	China	Retrospective study	Beyond LT criteria	6, 6/0	DCD	58 (range 50–62)	Camrelizumab (2) Sintilimab (2) Nivolumab (1) Pembrolizumab (1)	Downstaging	5.5 (range 1–20)	19.5 (range 12–45) days	11.9 (range 8.2~27.3) months
Qiao et al. (2021) (22)	China	Retrospective study	NR	7, 7/0	NR	53 ± 12.1	Pembrolizumab or camrelizumab	Downstaging	3 (1–5)	1.3 months	NR

Figures are number (percentage), median (interquartile range or range), or mean ± standard deviation.

NR, not reported; DD, deceased donor; LD, living donor; DBD, donation after brain death; DCD, donation after cardiac death.

There were one RCT, two retrospective cohort studies, and five retrospective studies. The median age of the patients ranged from 50.5 to 58 years. Approximately 90% of the patients were men. Four studies used only deceased donors, while two used both deceased and living donors. Five studies investigated the use of ICIs for downstaging treatment in patients with HCC beyond the LT criteria (13, 16, 20, 21). Three studies explored the application of ICIs for the downstaging or bridging treatment in patients with HCC who exceeded the LT criteria (14, 15, 19). The median number of ICI cycles prior to LT spanned from 3 to 7.5, with the median period ranging from 19.5 to 64 days. The median follow-up duration ranged from 8.1 to 33.4 months.

### The risk of bias of the included studies

One randomized controlled study was assessed using the JBI Critical Appraisal Checklist for RCTs, which was rated “unclear” for Q13. Two retrospective cohort studies were assessed using the JBI Critical Appraisal Checklist for Cohort Studies, which was rated “no” for IV and V. Five retrospective studies were assessed using the JBI Critical Appraisal Checklist for Case Series, which contains 10 items that assess the quality of case reports based on the selection of cases, disease or health problem evaluation, and presentation of case data. The details of the quality assessment are presented in **Table 2**.

**Table 2:** Quality assessment of the included studies.

JBI critical appraisal checklist for randomized controlled trials														
Study	Q1	Q2	Q3	Q4	Q5	Q6	Q7	Q8	Q9	Q10	Q11	Q12	Q13	Overall appraisal
Lv et al. (2024) (16)	Y	Y	Y	Y	Y	Y	Y	Y	Y	Y	Y	Y	**U**	Included
JBI critical appraisal checklist for cohort studies for included cohort studies														
Study	I	II	III	IV	V	VI	VII	VIII	IX	X	XI			
Lu et al. (2024) (17)	Y	Y	Y	N	N	Y	Y	Y	Y	Y	Y			Included
Wang et al. (2023) (18)	Y	Y	Y	N	N	Y	Y	Y	Y	Y	Y			Included
JBI critical appraisal checklist for case series for included retrospective studies														
Study	1	2	3	4	5	6	7	8	9	10				
Xu et al. (2024) (13)	Y	Y	Y	Y	Y	Y	Y	Y	N	Y				Included
Guo et al. (2024) (14)	Y	Y	Y	Y	Y	Y	Y	Y	N	Y				Included
Tabrizian et al. (2024) (15)	Y	Y	Y	Y	Y	N	Y	Y	N	Y				Included
Duan et al. (2022) (21)	Y	Y	Y	Y	Y	Y	Y	Y	N	NA				Included
Qiao et al. (2021) (22)	N	Y	Y	Y	Y	Y	Y	Y	N	NA				Included

Numbers Q1–Q13 in the heading signify the following: Q1, Was true randomization used for assignment of participants to treatment groups? Q2, Was allocation to treatment groups concealed? Q3, Were treatment groups similar at the baseline? Q4, Were participants blind to treatment assignment? Q5, Were those delivering the treatment blind to treatment assignment? Q6, Were treatment groups treated identically other than the intervention of interest? Q7, Were outcome assessors blind to treatment assignment? Q8, Were outcomes measured in the same way for treatment groups? Q9, Were outcomes measured in a reliable way? Q10, Was follow-up complete, and if not, were differences between groups in terms of their follow-up adequately described and analyzed? Q11, Were participants analyzed in the groups to which they were randomized? Q12, Was appropriate statistical analysis used? Q13, Was the trial design appropriate and any deviations from the standard RCT design (individual randomization, parallel groups) accounted for in the conduct and analysis of the trial?

Numbers I–XI in the heading signify the following: I, Were the two groups similar and recruited from the same population? II, Were the exposures measured similarly to assign people to both exposed and unexposed groups? III, Was the exposure measured in a valid and reliable way? VI, Were confounding factors identified? V, Were strategies to deal with confounding factors stated? VI, Were the groups/participants free of the outcome at the start of the study (or at the moment of exposure)? VII, Were the outcomes measured in a valid and reliable way? VIII, Was the follow-up time reported and sufficient to be long enough for outcomes to occur? XI, Was follow-up complete, and if not, were the reasons to loss to follow-up described and explored? X, Were strategies to address incomplete follow-up utilized? XI, Was appropriate statistical analysis used?

Numbers 1–10 in the heading signify the following: 1, Were there clear criteria for inclusion in the case series? 2, Was the condition measured in a standard, reliable way for all participants included in the case series? 3, Were valid methods used for identification of the condition for all participants included in the case series? 4, Did the case series have consecutive inclusion of participants? 5, Did the case series have complete inclusion of participants? 6, Was there clear reporting of the demographics of the participants in the study? 7, Was there clear reporting of clinical information of the participants? 8, Were the outcomes or follow-up results of cases clearly reported? 9, Was there clear reporting of the presenting site(s)/clinic(s) demographic information? 10, Was statistical analysis appropriate?

Y, yes; U, unclear; N, no; NA, not applicable.

### Allograft rejection

All eight included studies documented the AR rate after LT. The immunosuppression regimens (**Table 3**) were reported in seven studies: the immunosuppressive maintenance regimens were all based on CNIs, whereas basiliximab was used for induction therapy in four of these studies (13, 14, 19, 20). Five studies (13–15, 19, 20) proposed their own definitions of AR, and three studies (14, 15, 22) further specified their classification of AR according to the updated Banff classification (23). Five studies (13, 14, 19, 20, 22) further recorded the severity of AR according to the rejection activity index (RAI, **Supplementary Table S1**).

**Table 3:** Overview of the immunosuppression regimen, diagnostic criteria, and treatment of allograft rejection.

References	Immunosuppression regimen		Diagnostic criteria of acute rejection		Treatment
	Induction	Maintenance	Liver biopsy	Clinical signs	
Xu et al. (2024) (13)	Basiliximab and steroids	Combination regimens consisting of two or three medications, including tacrolimus, mycophenolate mofetil, steroids, and sirolimus	Biopsy-proven (*n* = 3)	–	One received steroid pulse therapy and ATG. One increased IS regimen dosage. One received steroid pulse therapy, IVIG, and plasmapheresis
Guo et al. (2024) (14)	Basiliximab and steroids	CNIs, antiproliferative agents, mTOR inhibitors, or steroids	Biopsy-proven (*n* = 7)	ALT or AST ≥2 times the upper limit and requiring treatment (*n* = 16)	All patients were treated by increased IS strength or high-dose steroids. Nine received therapy with IVIG, two received basiliximab, one received ATG, and one received plasmapheresis
Tabrizian et al. (2024) (15)	Steroids	Tacrolimus, mycophenolate mofetil, and methylprednisolone	Biopsy-proven (*n* = 7)	–	A combination of thymoglobulin, IVIG, plasmapheresis, rituximab, and steroids
Lv et al. (2024) (16)	NR	Tacrolimus, mycophenolate mofetil, and methylprednisolone	–	–	There are no cases of AR
Lu et al. (2024) (19)	Basiliximab and steroids	Alone or in combination with CNIs, mTOR inhibitors, or mycophenolate mofetil	Biopsy-proven (*n* = 5)	ALT or AST ≥1.5 times the upper limit for 48 h (*n* = 4)	Enhanced IS, including intensified CNIs and intravenous administration of steroids, basiliximab, and ATG, alone or in combination
Wang et al. (2023) (20)	Basiliximab	Tacrolimus combined with mycophenolate mofetil or sirolimus	Biopsy-proven (*n* = 4)	Elevation of transaminase during the recovery of liver function after LT or ALT or AST ≥2 times the upper limit and requiring treatment (*n* = 5)	Enhanced IS
Duan et al. (2022) (21)	Methylprednisolone	Tacrolimus, mycophenolate mofetil and prednisolone	–	–	There are no cases of AR
Qiao et al. (2021) (22)	NR	NR	–	–	NR

CNIs, calcineurin inhibitors; mTOR, mammalian target of rapamycin; AST, aspartate aminotransferase; ALT, alanine aminotransferase; IS, immunosuppression; ATG, antithymocyte globulin; IVIG, intravenous immunoglobulin; NR, not reported.

**Figure 2:**
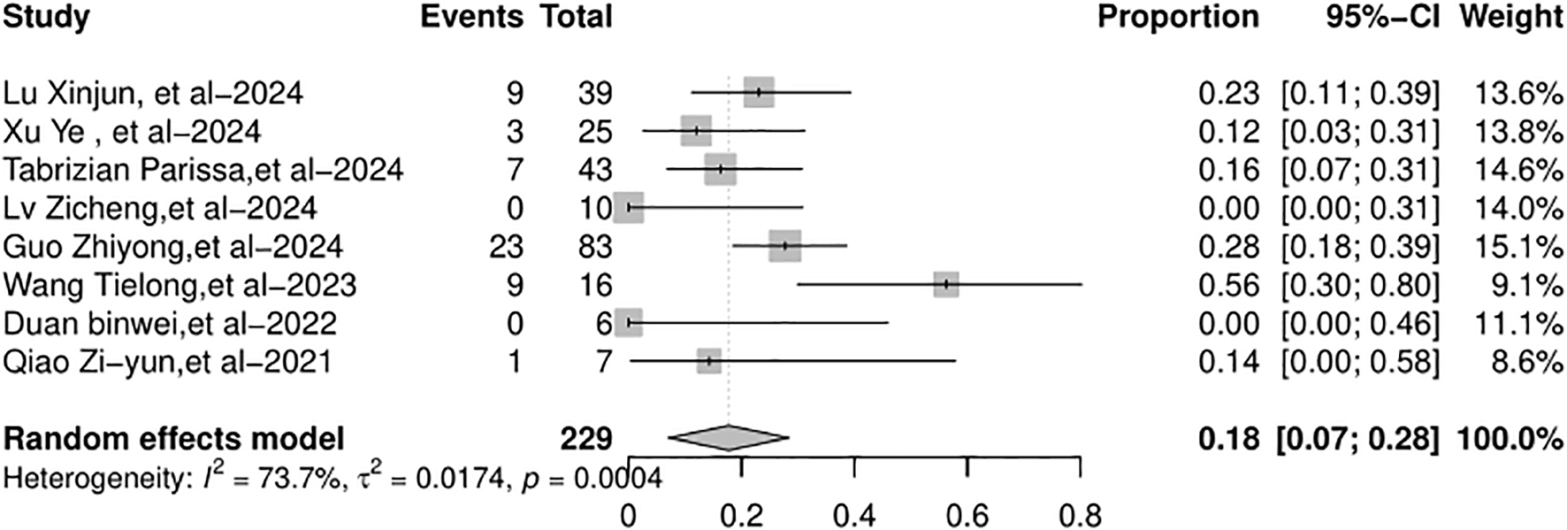
Forest plot on AR rate after liver transplantation. AR, allograft rejection.

**Figure 3:**
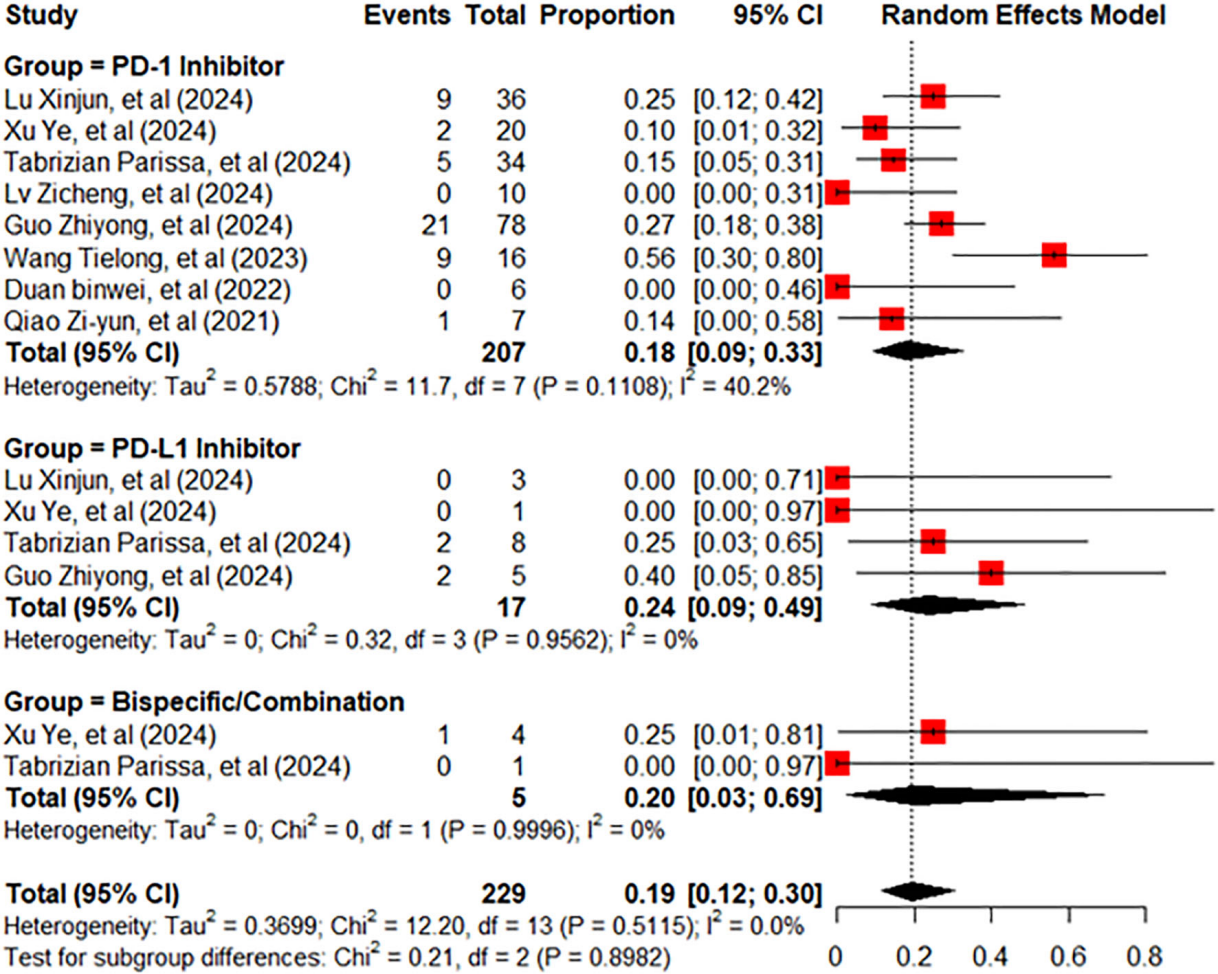
Forest plot showing changes in AR rate after liver transplantation by drug type. AR, allograft rejection.

Rejection occurred within 4–150 days post-transplant (**Supp** **lementary Table S1**). The AR rates across the studies varied from 0% to 56%. The random-effects model was used because of the significant heterogeneity (*I* ^2^ = 73.7%, *p* = 0.0004, **Figure 2**). Due to the substantial heterogeneity observed, a subgroup analysis was conducted by categorizing studies according to the type of ICIs used: PD-1 inhibitors, PD-L1 inhibitors, and bispecific/combination therapies. This stratification resulted in a remarkable reduction in heterogeneity, with the overall *I*² decreasing from 73.7% to 0% (**Figures 2**, **3**). This indicates that the considerable variation between studies was primarily attributable to differences in the type of therapeutic agents used. The pooled incidence of AR was 18% [95% confidence interval (95% CI): 9%–33%] in the PD-1 inhibitor group, with moderate heterogeneity (*I*² = 40.2%, *p* = 0.11). In the PD-L1 inhibitor group, the AR incidence was 24% (95% CI: 9%–49%), with no detectable heterogeneity (*I*² = 0%). The bispecific/combination therapy group exhibited an AR incidence of 20% (95% CI: 3%–69%). Although the point estimates of AR incidence varied slightly across the three subgroups (18%, 24%, and 20%, respectively), statistical testing indicated that these differences were not significant (*p* = 0.8982). This suggests that there may be no substantial difference in the incidence of AR between the different types of ICIs. The total AR rate was 19% (95% CI: 12%–30%).

All of the included studies provided data on the treatment of AR (**Table 3**). All recipients with AR were treated by enhanced immunosuppression, including intensified oral regimens and intravenous administration of steroids, basiliximab, antithymocyte globulin (ATG), intravenous immunoglobulin (IVIG), plasmapheresis, and rituximab, alone or in combination (**Table 3**).

**Figure 4:**
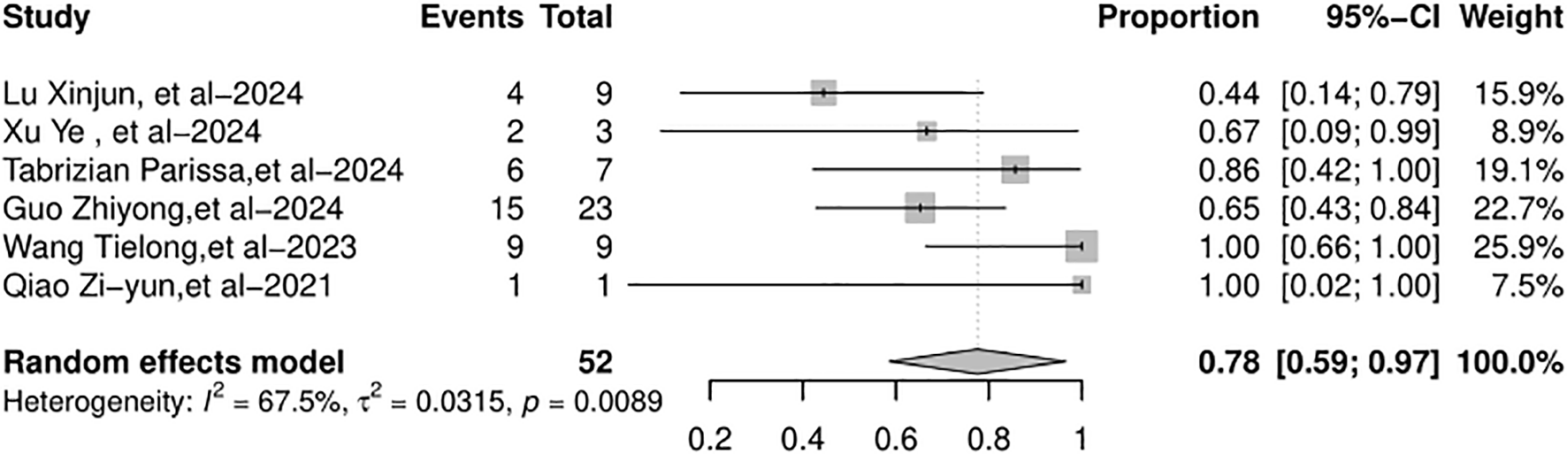
Forest plot on the full recovery rate of patients with AR. AR, allograft rejection.

**Figure 5:**
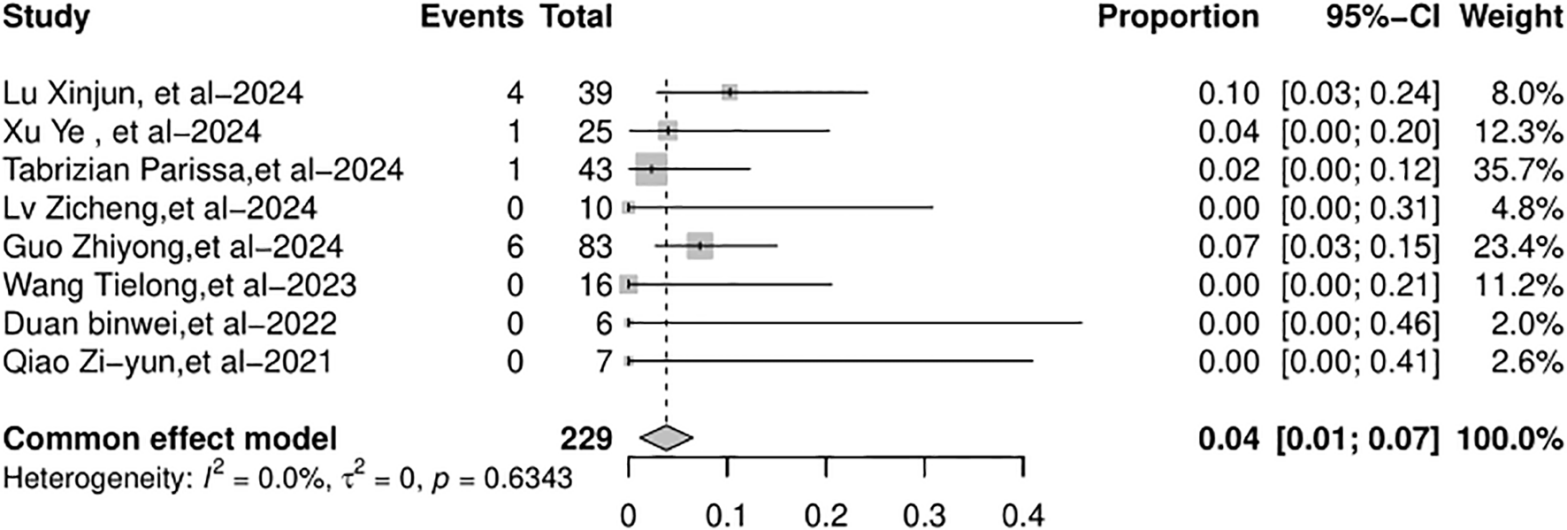
Forest plot presenting the pooled results regarding graft loss.

The full recovery rate across the studies varied from 44% to 100%. The random-effects model was used because of the significant heterogeneity (*I* ^2^ = 67.5%, *p* = 0.0089). The full recovery rate of the patients with AR (**Figure 4**) was 78% (95% CI: 59%–97%). The graft loss varied from 0% to 10%. The common-effects models were used and the graft loss (**Figure 5**) was 4% (95% CI: 1%–7%).

### Hepatocellular carcinoma recurrence

**Figure 6:**
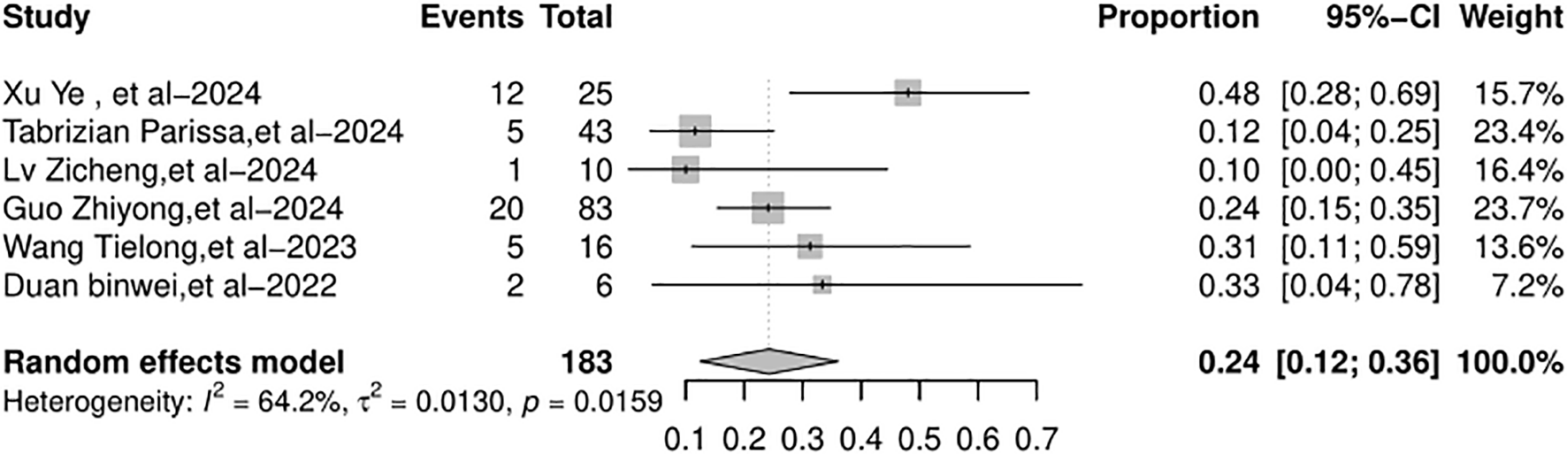
Forest plot of the pooled results of HCC recurrence rate. HCC, hepatocellular carcinoma.

Six studies included in the meta-analysis reported the HCC recurrence rate after LT. The HCC recurrence rate across the studies varied from 10% to 48%. The random-effects model was used because of the significant heterogeneity (*I* ^2^ = 64.2%, *p* = 0.0159). The overall HCC recurrence rate (**Figure 6**) was 24% (95% CI: 12%–36%).

### Survival

**Figure 7:**
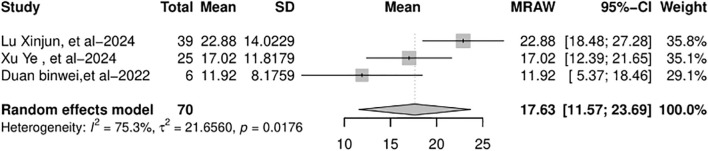
Forest plot of the pooled results of recurrence-free survival. RFS, recurrence-free survival.

RFS data were available in four out of eight studies, although the median RFS was statistically reached in only three of them. When the data were pooled, there was high heterogeneity and the funnel plot appeared symmetric. In the random-effects model (*I* ^2^ = 75.3%, *p* = 0.0176), the pooled median RFS was 17.63 months (95% CI: 11.57–23.69 months), as shown in **Figure 7**.

**Figure 8:**
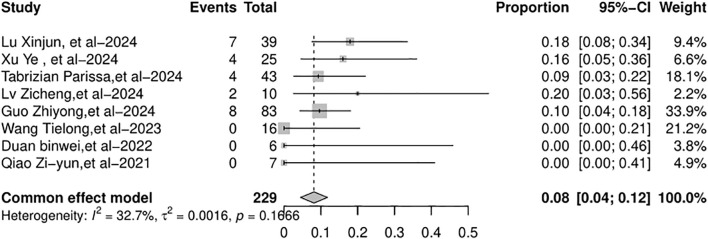
Forest plot of the pooled results of overall mortality.

**Figure 9:**
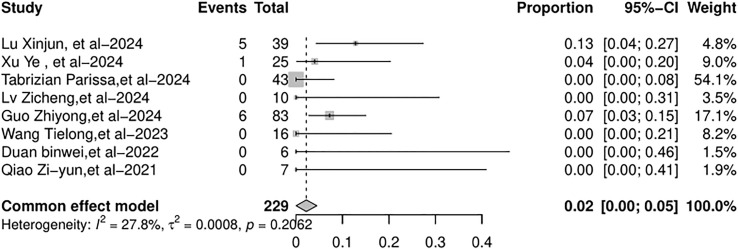
Forest plot of the pooled results of AR-related mortality. AR, allograft rejection.

All eight studies included in the meta-analysis documented the overall mortality and AR-related mortality. Common-effects models were used. The overall mortality rate was 8% (95% CI: 4%–12%), as shown in **Figure 8**. The AR-related mortality was 2% (95% CI: 0%–5%), as shown in **Figure 9**.

### Sensitivity analysis

Sensitivity analysis was performed by sequentially excluding one study at a time to evaluate the impact of each individual study on the pooled outcomes. The findings of this analysis clearly demonstrated that none of the pooled results with 95% CIs were substantially affected by any individual study. This indicated that the results of this meta-analysis were relatively reliable. The results of the sensitivity analysis are presented in **Supplementary Figure S1**.

### Publication bias

To ensure the validity of the meta-analysis results, funnel plots (**Supplementary Figure S2**) were used to estimate the publication bias. We considered that the publication bias exists for the AR rate, the full recovery rate of AR, graft loss, HCC recurrence rate, RFS, mortality, and AR-related mortality.

## Discussion

As the global prevalence and mortality rates of HCC have been increasing significantly, there is an urgent need for further advancements in treatment and management approaches. In recent years, ICI therapy has shown great promise for the treatment of HCC by reducing the mortality associated with the disease. In particular, the use of ICIs prior to LT is of great concern. For patients with HCC within the MC who respond to LRTs and undergo prompt LT, the probability of post-LT recurrence is so low that the consideration of immunotherapy lacks justification (5). For HCC patients within the MC, if they do not respond to LRT, need repeated LRT due to extended waiting times, or do not meet the LRT eligibility criteria, ICIs as a bridging treatment emerge as an appealing alternative (5, 15). For patients with HCC beyond the LT criterion, ICIs as a downstaging treatment are highly attractive, hoping to increase the proportion of patients who can receive LT and prolong patient survival (5, 15). The successful incorporation of ICIs into pre-LT downstaging or bridging therapy is supported by effective tumor size reduction within the LT criteria in a previous study (15). Complementing this, there is an impressive 3-year intention-to-treat survival rate of 71.1%, a post-LT survival rate of 85%, and a lack of high-grade adverse events during the waitlist period (15). Although the use of ICIs as downstaging or bridging therapies for LT in HCC patients is rapidly increasing, evidence regarding the feasibility and safety of ICI treatment prior to LT remains limited and controversial. In recent years, several new retrospective studies and RCT on the use of ICIs prior to LT have been published. We conducted this meta-analysis to comprehensively investigate the feasibility and safety of ICI treatment before LT.

Currently, post-LT AR is the main concern when ICIs are used for pre-LT treatment. It has been reported that the use of ICIs prior to LT may result in severe AR and subsequent graft loss (24, 25). The incidence of AR among patients receiving pre-LT ICI therapy varies across different studies, ranging from 0% to 56% (13–16, 19–22), whereas the incidence of AR is 10% to 30% in recipients not receiving ICIs (26, 27). The PLENTY pilot study showed that neither group experienced AR after LT, which is the only RCT that evaluated pre-LT ICI treatment in recipients diagnosed with HCC beyond MC (16). To our knowledge, our systematic review included the largest number of patients treated with ICIs prior to LT. The incidence of AR in our study was 19%. The full recovery rate of patients with AR was 78% and graft loss was 4%. Graft failure was reported in 6.3% at 6 months and 7.9% at 1 year for adult LT recipients not receiving ICIs in 2022 (27). The overall mortality rate was 8% and the AR-related mortality rate was 2% in our study. The overall mortality rate was reported to be 5.0% at 6 months and 6.5% at 1 year among adult LT recipients who did not receive ICIs by 2022 (27). From the above data, it appears that the incidence of AR and mortality among patients receiving ICIs is not higher than that among those who do not receive ICIs. However, it should be noted that the studies on the use of ICIs for pre-LT treatment have a limited number of participants, and the results have certain limitations. Regarding the impact of pre-LT use of ICIs on rejection, Tabrizian et al. mentioned that even when ICIs are used, there are many unique factors in the liver and during LT that can reduce the risk of AR (28): 1) Liver transplantation surgery usually involves significant blood loss, which, to a certain extent, clears the circulating ICIs. 2) Most significantly, when the liver is reperfused, extensive immunosuppression is initiated, which halts T-cell responses. 3) The liver possesses remarkable regenerative capacity, enabling it to recover from injuries. 4) The expression of major histocompatibility complex (MHC) class II antigens is rather feeble in the liver (5, 28).

The washout period of ICIs may be related to AR risk (11, 12). The importance of the washout period prior to LT and how long this washout period should last remain unclear. Additionally, the timing of LT is uncertain, making it extremely difficult to specify the exact washout period. Most studies have suggested that a time interval of 1 to 3 months is relatively safe (12, 15, 29). As a pilot study for a randomized controlled trial, PLENTY specified a 6-week washout period. No cases of rejection were observed among the enrolled patients. Wang et al. revealed a significant difference in the washout period between the rejection group and the non-rejection group (20). The median washout period in the rejection group was 21 days (15.5–27.5), while in the non-rejection group, it was 60 days (24-167) (20). Guo et al. demonstrated that the washout period of ICIs longer than 30 days was an independent protective factor against allograft rejection (14). An individual patient data meta-analysis revealed that the median washout period for patients with a ≤20% probability of allograft rejection was 94 (196) days (11). Kuo et al. discovered that a 1.5-fold half-life was the shortest safe washout period correlated with significant rejection-free survival (30). Although the appropriate length remains to be determined, a washout period prior to LT may be necessary. Different ICIs have varying half-lives and receptor occupancy levels, and the required safe washout periods before LT may also differ. ICIs are monoclonal antibodies that persist in the body for a long time after administration. The shortest half-life was 5 days (camrelizumab), whereas the longest extended beyond 20 days (pembrolizumab) (31–34). All ICIs bind to their targets with high affinity. In patients with advanced solid tumors, the occupancy of camrelizumab on PD-1 remained durable for at least 28 days following a single infusion at doses of 200 and 400 mg (35). The PD-1 occupancy of nivolumab only begins to decay 85 days after administration at a dose of 10 mg/kg (36). Considering the pharmacokinetic properties and clinical experience of different ICIs, it is advisable to develop more precise and individualized guidelines for the washout period prior to transplantation. For certain ICIs with a shorter half-life, a relatively brief washout period may suffice, whereas those with a longer half-life are likely to require an extended washout duration. Currently, there is no available pharmacokinetic data before and after LT. Blood loss and resuscitation during surgery may result in a drastic reduction in serum levels. Further research is still needed to address this aspect.

Apart from the period, some researchers have mentioned that different ICI therapies prior to LT also vary in terms of the risk of post-transplantation rejection (5). The immune targets of ICIs include the PD-1 and its ligand, PD-L1, and CTLA-4. The commonly used PD-1 inhibitors include camrelizumab, sintilimab, pembrolizumab, nivolumab, toripalimab, tislelizumab, and penpulimab. Envafolimab, atezolizumab, and durvalumab are PD-L1 inhibitors, while ipilimumab is a CTLA-4 inhibitor. The PD-1/PD-L1 interaction, which promotes Treg development and maintenance, suppresses T-cell activation, and causes T-cell exhaustion, is crucial for inducing and maintaining solid organ tolerance (37). In contrast to control wild-type mice, PD-1^−/−^ or PD-L1^−/−^ recipient mice rejected cardiac allograft transplantation, even when immunosuppressive treatment was administered (38). Similarly, in a mouse model of LT, blocking the PD-1/PD-L1 pathway with anti-PD-L1 antibodies or using PD-L1 knockout mice as donors resulted in graft rejection (39). These experiments indicate that the expression of PD-L1 on both the recipient’s cells and graft cells is crucial for maintaining graft acceptance. Although PD-1 and PD-L1 inhibitors have often been used interchangeably, evidence indicates that alloimmune responses (i.e., rejection) are more likely to occur with anti-PD-L1 agents than with anti-PD-1 agents (5). This is because PD-L1, aside from being the ligand for PD-1, also serves as a ligand for the B7-1 (CD 80) checkpoint (12). Compared with PD-1/PD-L1, CTLA-4 has less impact on allograft acceptance (40). Our analysis revealed that the incidence of AR was 24% in the PD-L1 inhibitor group, 18% in the PD-1 inhibitor group, and 20% in the bispecific/combination therapies group. Although numerically higher in the PD-L1 group compared to the PD-1 group, this difference did not reach statistical significance. None of the studies included in our analysis involved patients treated with CTLA-4 inhibitors alone. Among the five patients in the bispecific/combination therapies group, one received cadonilimab (a PD-1/CTLA-4-bispecific antibody), three were treated with a combination of PD-1 and PD-L1 inhibitors, and one received a combined PD-L1 and CTLA-4 inhibitor. The impact of pre-LT application of different ICIs on post-transplantation rejection still requires further in-depth exploration. Currently, there is only one case series regarding the pre-LT use of PD-L1 inhibitors (atezolizumab–bevacizumab) in patients with HCC. In this series, none of the five patients experienced recurrence or rejection after the surgery (41). There are four case series regarding pre-LT use of PD-1 inhibitors (nivolumab) in HCC patients (**Supplementary Table S2**) (42–45). In one study, none of the five patients experienced rejection (43). In the remaining three studies, cases of rejection were reported, and the washout periods of ICIs in all these cases were less than 90 days (41–43). Future studies should focus on comparing the efficacy and safety profiles of different ICIs—including PD-1 inhibitors, PD-L1 inhibitors, and CTLA-4 inhibitors—in the neoadjuvant setting prior to liver transplantation. Such head-to-head comparative studies are of great significance, as they can provide definitive evidence for identifying the optimal immunotherapeutic strategy, thereby balancing the potential antitumor efficacy against the risk of adverse events, including allograft rejection.

The management of post-LT immunosuppression differs across various centers (13–16, 19–22). The use of high-dose steroids for immune induction in LT widely suppresses the immune response. Specifically, the proliferation of T cells and T-cell apoptosis almost cease immediately. Given the benefits of reducing steroid use in LT for HCC, the steroid dosages in the immune induction regimens for LT currently vary across different centers. High-dose steroids actually serve as the treatment for severe ICI-related immune reactions (46, 47). It is possible that the variability in the intensity of early-stage steroid dosing explains some of the differences in the reported incidence of AR among patients who received ICIs prior to LT. The induction of immunosuppression using T-cell-depleting agents, such as ATG, leads to a substantial depletion of T cells. This could be an efficient means to prevent ICI-related rejection. Given the use of ICIs prior to LT, research on immunosuppression management, especially in terms of immune induction schemes, is limited, and there is currently no consensus on the optimal plan. However, it is clear that a stronger immunosuppression regimen should be used in patients with pre-LT use of ICIs to guard against allograft rejection.

Emerging evidence suggests that biomarkers could enhance the prediction of rejection risk in patients receiving ICIs prior to LT. Tabrizian et al. mentioned that a possible explanation for the sporadic cases of severe rejection observed could be the presence of immunological memory against the alloantigens presented in the liver graft (5). This is supported by observations that ICI-responsive cancer patients often harbor pre-existing memory T cells targeting tumor antigens (48). The enzyme-linked immunospot (ELISpot) assay could be employed to detect donor-reactive memory T cells through interferon-γ (IFN-γ) secretion, providing a functional measure of alloreactive potential (49). If such pre-existing memory T-cell responses are conclusively linked to post-transplant rejection in ICI-treated patients, IFN-γ-based assays may offer a practical tool for risk stratification. Furthermore, Qiao et al. reported that patients who experienced acute rejection after pre-LT ICI exposure showed rapid increases in the CD4/CD8 ratio and CD8^+^CD3^+^ T-cell counts within 5 days post-transplant (22), suggesting that early immune monitoring could aid rejection prediction. Additionally, existing research evidence has demonstrated a positive correlation between the expression levels of PD-L1 and PD-1 and the severity of post-transplant rejection in the fields of heart and corneal transplantation. This suggests that the expression levels of PD-L1 and PD-1 in the graft hold potential value as predictive indicators for acute rejection. However, clinical studies investigating this correlation in the context of liver transplantation are currently limited by small sample sizes (50, 51). Therefore, it is proposed that future multicenter, large-cohort studies are needed to further validate the predictive efficacy of graft PD-L1 and PD-1 expression levels for acute rejection in liver transplantation. In summary, these findings highlight the potential utility of integrated biomarker platforms—which incorporate cellular, soluble molecular, and transcriptional profiling—in refining pre- and post-transplant risk assessment, enhancing the identification and management of high-risk patients, and supporting the personalized management of ICI-related rejection.

Besides rejection, long-term oncologic outcomes represent a particular concern for patients who are undergoing ICI treatment prior to LT. Our study showed that the HCC recurrence rate was 24% and the pooled median RFS was 17.63 months. In a large-scale multicenter study, the recurrence rate of HCC after LT was 10% among patients within the MC (4). For patients who met the MC criteria after successful downstaging, the HCC recurrence rate after LT reached 15.8%. In contrast, for patients who exceeded the MC criteria, the recurrence rate was as high as 35.2% (4). In the patients included in our study, ICIs were mainly used for downstaging treatment. At the time of LT, some patients met the LT criteria, while others still exceeded them. Therefore, the recurrence rate in our study was higher than that of patients who have successfully undergone downstaging treatment. Additionally, Rezaee-Zavareh et al. mentioned that the number of ICI cycles and tumor burden are likely to exert an impact on the recurrence risk (11). Fewer ICI cycles and a high tumor burden are related to a higher risk of recurrence (11). During the short follow-up period, the rates of post-LT HCC recurrence among patients with complete response (CR), partial response (PR), stable disease (SD), and progressive disease (PD) were 0%, 19.5%, 37.0%, and 33.3%, respectively (14). Therefore, Guo et al. reported that transplantation can be performed in patients with CR or PR after undergoing downstaging treatment with ICIs prior to LT. Nevertheless, caution should be exercised when considering LT in patients with PD or SD (14).

ICIs may cause various systemic adverse events during antitumor treatment, and patients awaiting liver transplantation often have underlying conditions like abnormal liver function and immune dysregulation. Thus, this study recommends the following: strengthening monitoring for major adverse events (e.g., hypersensitivity reactions, infections, bleeding) and developing targeted management strategies to enhance treatment safety in future clinical practice and research. 1) Monitoring system construction a) core mechanism: Establish a multidimensional, full-cycle assessment framework. b) Pre-ICI screening: Comprehensively evaluate patients’ underlying diseases (e.g., allergic disease history, chronic infections, coagulation disorders) and review past medical history to predict ICI-related risks (52, 53). Conduct baseline tests (blood routine, liver/kidney function, inflammatory markers, infectious disease screening, coagulation function) to exclude high-risk ineligible patients. c) During ICI monitoring: Shorten monitoring intervals (repeat lab tests every 2 weeks) and combine regular physical exams, imaging (chest CT for infections, abdominal imaging for bleeding risks), and patient self-reports (e.g., rash, fever, dyspnea) to detect early adverse event signs. Prioritize hypersensitivity reactions: acute reactions (anaphylactic shock, laryngeal edema, severe rash) within minutes to hours post-administration and delayed reactions (maculopapular rash, pruritus with mucosal involvement) 1–2 weeks later (43). Cover both common bacterial infections (e.g., pneumonia, urinary tract infection) and opportunistic infections (e.g., cytomegalovirus pneumonia, fungal infection) for infection monitoring. Focus on gastrointestinal bleeding (melena, hematemesis), intra-abdominal bleeding, and puncture-site bleeding for bleeding monitoring; regularly test prothrombin time, INR, and platelet count; and assess risk factors (active ulcers, portal hypertension). 2) Management strategy formulation a) guiding principle: Follow “stratified management and precision intervention”; adopt a stepped protocol based on adverse event severity (per CTCAE criteria). b) Stratified responses: grade 1–2 mild events (e.g., mild rash, low-grade fever): Continue ICI treatment under close monitoring with symptomatic care. Grade 3–4 severe events (e.g., anaphylactic shock, severe pneumonia, massive gastrointestinal bleeding): Immediately discontinue ICIs and initiate emergency treatment. c) Multidisciplinary collaboration: Establish an adverse event emergency team (integrating hepatology, transplant surgery, infectious diseases, and allergy/immunology). This enables prompt multidisciplinary consultations, individualized plans, minimized impact on patient prognosis, and enhanced safety of pre-liver transplantation ICI therapy.

There are still numerous issues to be resolved regarding pre-LT ICI therapy for HCC: 1) Patient selection: patients who exceed LT criteria and have achieved a favorable response after ICI treatment may gain more benefits after LT; 2) timing: maintaining a washout period of 1 to 3 months may be relatively safe. The washout periods required for different ICIs may vary, as they differ in terms of response rates, half-lives, and target occupancy times; 3) selection of ICIs: the type, dosage, and number of ICI cycles, along with whether they are used singly or in combination for bridging or downstaging, can also impact outcomes; 4) development of biomarkers: develop biomarkers such as graft PD-L1 expression, tumor mutation burden, tumor-infiltrating lymphocytes, T-cell ratio, and IL-6 as predictive factors for ICI response and allograft rejection, and integrate these markers with artificial intelligence models (54, 55); 5) the multidisciplinary team: in pre-LT ICI treatment for HCC patients, the multidisciplinary team (MDT) should be involved throughout the process, including recipient assessment, medication regimen formulation, efficacy evaluation, and monitoring, prevention, and treatment of adverse reactions. The MDT management model can provide recipients with a more scientific, reasonable, and comprehensive treatment plan, thereby increasing the treatment benefits for recipients.

There are several limitations in our study that should be acknowledged. First, the majority of included studies were retrospective in design, with relatively small sample sizes and a lack of control groups, which limits the ability to draw definitive conclusions regarding efficacy and risk assessments. Second, the potential for publication bias must be considered, as studies with positive outcomes are more likely to be published, while negative results may be underrepresented. Third, significant heterogeneity was observed across the studies, which may stem from variations in treatment intent (e.g., downstaging vs. bridging), the timing of ICI administration relative to liver transplantation, and the use of concurrent or prior locoregional therapies. Although planned subgroup analyses were conducted to explore sources of heterogeneity, the limited number of studies constrained a thorough investigation. Additionally, approximately half of the reported rejection cases were not biopsy-confirmed, making it difficult to definitively attribute these events to ICI use. The follow-up duration in most studies was relatively short, and data on long-term outcomes such as overall survival were often unavailable. Furthermore, as most patients received additional treatments prior to transplantation (e.g., tyrosine kinase inhibitors), it remains challenging to isolate the specific contribution of ICIs to post-transplant outcomes. Although locoregional therapy did not significantly influence the primary outcomes such as graft rejection, its interplay with ICIs warrants further investigation.

## Conclusion

The results of the current study indicate that the use of ICIs for bridging or downstaging in the treatment of HCC prior to liver transplantation is feasible. To mitigate the risk of allograft rejection effectively, a washout period spanning 1–3 months is considered reasonable. This meta-analysis calls for well-designed prospective RCTs to evaluate the efficacy and safety of different ICIs in the bridging or downstaging treatment of HCC prior to LT.

## Data Availability

The original contributions presented in the study are included in the article/**Supplementary Material**. Further inquiries can be directed to the corresponding author.
