# Supplementary material for: Navigating the risks: a systematic review of immune checkpoint inhibitor therapy before liver transplant for hepatocellular carcinoma and its impact on allograft rejection and survival outcomes
Source: Front Oncol. 2025 Oct 29;15:1689820. doi: 10.3389/fonc.2025.1689820 (PMC12605506; doi:10.3389/fonc.2025.1689820)
Supplement: Supplementary file 1 [file DataSheet1.docx]

1. **Pubmed 62**

#1 **Carcinoma, Hepatocellular[MeSH Terms]**

#2 **((((((((((((((Carcinomas, Hepatocellular[Title/Abstract]) OR (Hepatoma[Title/Abstract])) OR (Hepatomas[Title/Abstract])) OR (Liver Cancer, Adult[Title/Abstract])) OR (Adult Liver Cancers[Title/Abstract])) OR (Cancer, Adult Liver[Title/Abstract])) OR (Cancers, Adult Liver[Title/Abstract])) OR (Liver Cancers, Adult[Title/Abstract])) OR (Liver Cell Carcinoma[Title/Abstract])) OR (Carcinoma, Liver Cell[Title/Abstract])) OR (Carcinomas, Liver Cell[Title/Abstract])) OR (Cell Carcinoma, Liver[Title/Abstract])) OR (Cell Carcinomas, Liver[Title/Abstract])) OR (Liver Cell Carcinomas[Title/Abstract])) OR (Liver Cell Carcinoma, Adult[Title/Abstract])**

#3 #1 OR #2

#4  Liver Transplantation [MeSH Terms]

#5 (((((Hepatic Transplantation[Title/Abstract]) OR (Hepatic Transplantations[Title/Abstract])) OR (Liver Transplantations[Title/Abstract])) OR (Liver Transplant[Title/Abstract])) OR (Liver Transplants[Title/Abstract])) OR (Grafting Liver[Title/Abstract])

#6 #4 OR #5

#7 #3 AND #6

#8 **Immune Checkpoint Inhibitors[MeSH Terms]**

#9 **(((((((((((((((((((((((((Immune Checkpoint Blockers[Title/Abstract]) OR (Immune Checkpoint Inhibitor[Title/Abstract])) OR (CTLA-4 Inhibitors[Title/Abstract])) OR (CTLA 4 Inhibitors[Title/Abstract])) OR (Cytotoxic T-Lymphocyte-Associated Protein 4 Inhibitors[Title/Abstract])) OR (Cytotoxic T Lymphocyte Associated Protein 4 Inhibitors[Title/Abstract])) OR (Cytotoxic T-Lymphocyte-Associated Protein 4 Inhibitor[Title/Abstract])) OR (Cytotoxic T Lymphocyte Associated Protein 4 Inhibitor[Title/Abstract])) OR (CTLA-4 Inhibitor[Title/Abstract])) OR (CTLA 4 Inhibitor[Title/Abstract])) OR (PD-1 Inhibitors[Title/Abstract])) OR (PD 1 Inhibitors[Title/Abstract])) OR (Programmed Cell Death Protein 1 Inhibitor[Title/Abstract])) OR (Programmed Cell Death Protein 1 Inhibitors[Title/Abstract])) OR (PD-1 Inhibitor[Title/Abstract])) OR (PD 1 Inhibitor[Title/Abstract])) OR (Immune Checkpoint Blockade[Title/Abstract])) OR (Immune Checkpoint Inhibition[Title/Abstract])) OR (PD-L1 Inhibitors[Title/Abstract])) OR (PD L1 Inhibitors[Title/Abstract])) OR (Programmed Death-Ligand 1 Inhibitors[Title/Abstract])) OR (Programmed Death Ligand 1 Inhibitors[Title/Abstract])) OR (PD-L1 Inhibitor[Title/Abstract])) OR (PD L1 Inhibitor[Title/Abstract])) OR (PD-1-PD-L1 Blockade[Title/Abstract])) OR (PD 1 PD L1 Blockade[Title/Abstract])**

#10 #8 OR #9

#11 **((((((atezolizumab[Supplementary Concept]) OR (anti-PDL1[Title/Abstract])) OR (MPDL3280A[Title/Abstract])) OR (MPDL-3280A[Title/Abstract])) OR (Tecentriq[Title/Abstract])) OR (RG7446[Title/Abstract])) OR (RG-7446[Title/Abstract])**

#12 **((((((((((Nivolumab[MeSH Terms]) OR (MDX-1106[Title/Abstract])) OR (MDX1106[Title/Abstract])) OR (MDX 1106[Title/Abstract])) OR (Opdivo[Title/Abstract])) OR (BMS-936558[Title/Abstract])) OR (BMS936558[Title/Abstract])) OR (BMS 936558[Title/Abstract])) OR (ONO-4538[Title/Abstract])) OR (ONO4538[Title/Abstract])) OR (ONO 4538[Title/Abstract])**

#13 **((((pembrolizumab[Supplementary Concept]) OR (MK-3475[Title/Abstract])) OR (Keytruda[Title/Abstract])) OR (lambrolizumab[Title/Abstract])) OR (SCH-900475[Title/Abstract])**

#14 **(((((((((Ipilimumab[MeSH Terms]) OR (Anti-CTLA-4 MAb Ipilimumab[Title/Abstract])) OR (Anti CTLA 4 MAb Ipilimumab[Title/Abstract])) OR (Ipilimumab, Anti-CTLA-4 MAb[Title/Abstract])) OR (MDX 010[Title/Abstract])) OR (MDX-010[Title/Abstract])) OR (MDX010[Title/Abstract])) OR (MDX-CTLA-4[Title/Abstract])) OR (MDX CTLA 4[Title/Abstract])) OR (Yervoy[Title/Abstract])**

#15  **((sintilimab[Supplementary Concept]) OR (IBI 308[Title/Abstract])) OR (IBI308[Title/Abstract])**

#16 **(((camrelizumab[Supplementary Concept]) OR (SHR-1210[Title/Abstract])) OR (SHR 1210[Title/Abstract])) OR (carrelizumab[Title/Abstract])**

#17 **(((durvalumab[Supplementary Concept]) OR (MEDI4736[Title/Abstract])) OR (MEDI-4736[Title/Abstract])) OR (Imfinzi[Title/Abstract])**

#**18 (tislelizumab[Supplementary Concept]) OR (BGB-A317[Title/Abstract])**

#19 #10 OR #11 OR #12 OR #13OR #14 OR #15OR #16OR #17OR #18

#20 #7 AND #19

1. **Embase 661**

#1 'liver cell carcinoma'/exp

#2 'carcinoma in the liver':ab,ti OR 'carcinoma of the liver':ab,ti OR 'carcinoma, hepatic cell':ab,ti OR 'carcinoma, hepatocellular':ab,ti OR 'carcinoma, liver':ab,ti OR 'carcinoma, liver cell':ab,ti OR 'hepatic carcinoma':ab,ti OR 'hepatic cell carcinoma':ab,ti OR 'hepato carcinoma':ab,ti OR 'hepato-cellular carcinoma':ab,ti OR hepatocarcinoma:ab,ti OR 'hepatocellular carcinoma':ab,ti OR 'hepatocellular carcinomata':ab,ti OR 'hepatocyte carcinoma':ab,ti OR 'hepatocytic carcinoma':ab,ti OR hepatoma:ab,ti OR hepatomata:ab,ti OR hepatomatous:ab,ti OR 'liver carcinoma':ab,ti OR 'liver carcinoma rupture':ab,ti OR 'malignant hepatoma':ab,ti OR 'primary liver carcinoma':ab,ti OR 'liver cell carcinoma':ab,ti

#3 #1 OR #2

#4'liver transplantation'/exp

#5'auxiliary liver transplantation':ab,ti OR 'hepatic transplantation':ab,ti OR 'liver heterotopic transplantation':ab,ti OR 'liver orthotopic transplantation':ab,ti OR 'liver tissue transplantation':ab,ti OR 'orthotopic liver transplantation':ab,ti OR 'transplantation, hepatic':ab,ti OR 'transplantation, liver':ab,ti OR 'liver transplantation':ab,ti

#6 #4 OR #5

#7 #3 AND #6

#8 'immune checkpoint inhibitor'/exp

#9 'immune checkpoint inhibitor':ab,ti OR 'immune checkpoint blockers':ab,ti OR 'immune checkpoint inhibitors':ab,ti OR 'ctla-4 inhibitors':ab,ti OR 'ctla 4 inhibitors':ab,ti OR 'cytotoxic t-lymphocyte-associated protein 4 inhibitors':ab,ti OR 'cytotoxic t lymphocyte associated protein 4 inhibitors':ab,ti OR 'cytotoxic t-lymphocyte-associated protein 4 inhibitor':ab,ti OR 'cytotoxic t lymphocyte associated protein 4 inhibitor':ab,ti OR 'ctla-4 inhibitor':ab,ti OR 'ctla 4 inhibitor':ab,ti OR 'pd-1 inhibitors':ab,ti OR 'pd 1 inhibitors':ab,ti OR 'programmed cell death protein 1 inhibitor':ab,ti OR 'programmed cell death protein 1 inhibitors':ab,ti OR 'pd-1 inhibitor':ab,ti OR 'pd 1 inhibitor':ab,ti OR 'immune checkpoint blockade':ab,ti OR 'immune checkpoint inhibition':ab,ti OR 'pd-l1 inhibitors':ab,ti OR 'pd l1 inhibitors':ab,ti OR 'programmed death-ligand 1 inhibitors':ab,ti OR 'programmed death ligand 1 inhibitors':ab,ti OR 'pd-l1 inhibitor':ab,ti OR 'pd l1 inhibitor':ab,ti OR 'pd-1-pd-l1 blockade':ab,ti OR 'pd 1 pd l1 blockade':ab,ti

#10 #8 OR #9

#11'atezolizumab'/exp

#12 'atezolizumab':ab,ti OR 'monoclonal antibody mpdl 3280a':ab,ti OR 'monoclonal antibody mpdl3280a':ab,ti OR 'mpdl 3280a':ab,ti OR mpdl3280a:ab,ti OR 'rg 7446':ab,ti OR rg7446:ab,ti OR 'ro 5541267':ab,ti OR ro5541267:ab,ti OR tecentriq:ab,ti OR tecntriq:ab,ti

#13 #11 OR #12

#14 'nivolumab'/exp

#15 'nivolumab':ab,ti OR 'ba 1104':ab,ti OR ba1104:ab,ti OR 'bms 936558':ab,ti OR bms936558:ab,ti OR 'cmab 819':ab,ti OR cmab819:ab,ti OR 'ly 01015':ab,ti OR ly01015:ab,ti OR 'mdx 1106':ab,ti OR mdx1106:ab,ti OR 'ono 4538':ab,ti OR ono4538:ab,ti OR opdivo:ab,ti OR 'pbp 2101':ab,ti OR pbp2101:ab,ti OR xdivane:ab,ti

#16 14# OR #15

#17 'pembrolizumab'/exp

#18 'pembrolizumab':ab,ti OR 'bcd 201':ab,ti OR 'bcd201':ab,ti OR 'keytruda':ab,ti OR 'lambrolizumab':ab,ti OR 'mk 3475':ab,ti OR 'mk3475':ab,ti OR 'pbp 2102':ab,ti OR 'pbp2102':ab,ti OR 'sch 900475':ab,ti OR 'sch900475':ab,ti OR 'xtrudane':ab,ti

#19 17# OR #18

#20 'ipilimumab'/exp

#21 'ipilimumab':ab,ti OR 'bms 734016':ab,ti OR bms734016:ab,ti OR 'cs 1002':ab,ti OR cs1002:ab,ti OR 'eb 1003':ab,ti OR 'hlx 13':ab,ti OR hlx13:ab,ti OR 'ibi 310':ab,ti OR ibi310:ab,ti OR 'mdx 010':ab,ti OR 'mdx 101':ab,ti OR mdx010:ab,ti OR mdx101:ab,ti OR 'pbp 1701':ab,ti OR 'pbp1701':ab,ti OR 'strentarga':ab,ti OR 'yervoy':ab,ti

#22 20# OR #21

#23'sintilimab'/exp

#24'sintilimab':ab,ti OR 'ibi 308':ab,ti OR 'ibi308':ab,ti OR 'tyvyt':ab,ti

#25 #23 OR #24

#26 'camrelizumab'/exp

#27'camrelizumab':ab,ti

#28 26# OR #27

#29 'durvalumab'/exp

#30 'durvalumab':ab,ti OR 'imfinzi':ab,ti OR 'medi 4736':ab,ti OR 'medi4736':ab,ti

#31 #29 OR #30

#32 'tislelizumab'/exp

#33'tislelizumab':ab,ti OR 'bgb a317':ab,ti OR  'bgba317':ab,ti OR 'bgn 1':ab,ti OR  'bgn1':ab,ti OR 'jhl 2108':ab,ti OR  'jhl2108':ab,ti OR  'tevimbra':ab,ti OR  'tilelizumab':ab,ti OR  'tirelizumab':ab,ti OR 'tizveni':ab,ti OR 'vdt 482':ab,ti OR  'vdt482':ab,ti

#34 #32 OR #33

#35 #10 OR #13 OR #16 OR #19 OR #22 OR #25 OR #28 OR #31 OR #34

#36 #7 AND #35

1. **Web of science 254**

**#1** **"Carcinoma Hepatocellular" OR "Carcinomas Hepatocellular" OR "Hepatoma" OR "Hepatomas" OR "Liver Cancer Adult" OR “Cancer Adult Liver” OR “Cancers Adult Liver” OR “Liver Cancers Adult” OR “Liver Cell Carcinoma” OR “Carcinoma Liver Cell” OR “Carcinomas Liver Cell” OR “Cell Carcinoma Liver” OR “Cell Carcinomas Liver” OR “Liver Cell Carcinomas” OR “Liver Cell Carcinoma”** (Topic)

**#2 “Liver Transplantation” OR “Hepatic Transplantation” OR “Hepatic Transplantations” OR “Liver Transplantations” OR “Liver Transplant” OR “Liver Transplants” OR “Grafting Liver”** (Topic)

**#3 #2 AND #1**

**#4“Immune Checkpoint Inhibitors” OR “Immune Checkpoint Blockers” OR “Immune Checkpoint Inhibitor” OR “CTLA-4 Inhibitors” OR “CTLA 4 Inhibitors” OR “Cytotoxic T-Lymphocyte-Associated Protein 4 Inhibitors” OR “Cytotoxic T Lymphocyte Associated Protein 4 Inhibitors” OR “Cytotoxic T-Lymphocyte-Associated Protein 4 Inhibitor” OR “Cytotoxic T Lymphocyte Associated Protein 4 Inhibitor” OR “CTLA-4 Inhibitor” OR “CTLA 4 Inhibitor” OR “PD-1 Inhibitors” OR “PD 1 Inhibitors” OR “Programmed Cell Death Protein 1 Inhibitor” OR “Programmed Cell Death Protein 1 Inhibitors” OR “PD-1 Inhibitor” OR “PD 1 Inhibitor” OR “Immune Checkpoint Blockade” OR “Immune Checkpoint Inhibition” OR “PD-L1 Inhibitors” OR “PD L1 Inhibitors” OR “Programmed Death-Ligand 1 Inhibitors” OR “Programmed Death Ligand 1 Inhibitors” OR “PD-L1 Inhibitor” OR “PD L1 Inhibitor” OR “PD-1-PD-L1 Blockade” OR “PD 1 PD L1 Blockade”**

**#5 “atezolizumab” OR “anti-PDL1” OR “MPDL3280A” OR “MPDL-3280A” OR “Tecentriq” OR “RG7446” OR “RG-7446”** (Topic)

**#6 “Nivolumab” OR “MDX-1106” OR “MDX1106” OR “MDX 1106” OR “Opdivo” OR “BMS-936558” OR “BMS936558” OR “BMS 936558” OR “ONO-4538” OR “ONO4538” OR “ONO 4538”** (Topic)

**#7“pembrolizumab” OR “MK-3475” OR “Keytruda” OR “lambrolizumab” OR “SCH-900475”** (Topic)

**#8“Ipilimumab” OR “Anti-CTLA-4 MAb Ipilimumab” OR “Anti CTLA 4 MAb Ipilimumab” OR “Ipilimumab Anti-CTLA-4 Mab” OR “MDX 010” OR “MDX-010” OR “MDX010” OR “MDX-CTLA-4” OR “MDX CTLA 4” OR “Yervoy”** (Topic)

**#9 “sintilimab” OR “IBI 308” OR “IBI308”** (Topic)

**#10“camrelizumab” OR “SHR-1210” OR “SHR 1210” OR “carrelizumab”** (Topic)

**#11“durvalumab” OR “MEDI4736” OR “MEDI-4736” OR “Imfinzi”** (Topic)

**#12“tislelizumab” OR “BGB-A317”** (Topic)

**#13 #4 OR #5 OR #6 OR #7 OR #8 OR #9 OR #10 OR #11 OR #12**

**#14 #3 AND #13**

1. **Cochrane 62**

#1 MeSH descriptor: [Carcinoma, Hepatocellular] explode all trees

#2 (Carcinoma, Hepatocellular)**:ti,ab,kw OR (Carcinomas, Hepatocellular):ti,ab,kw OR (Hepatoma):ti,ab,kw OR (Hepatomas):ti,ab,kw OR (Liver Cancer, Adult):ti,ab,kw OR (Adult Liver Cancers):ti,ab,kw OR (Cancer, Adult Liver):ti,ab,kw OR (Cancers, Adult Liver):ti,ab,kw OR (Liver Cancers, Adult):ti,ab,kw OR (Liver Cell Carcinoma):ti,ab,kw OR (Carcinoma, Liver Cell):ti,ab,kw OR (Carcinomas, Liver Cell):ti,ab,kw OR (Cell Carcinoma, Liver):ti,ab,kw OR (Cell Carcinomas, Liver):ti,ab,kw OR (Liver Cell Carcinomas):ti,ab,kw OR (Liver Cell Carcinoma, Adult):ti,ab,kw**

#3 #1 OR #2

#4 MeSH descriptor: [Liver Transplantation] explode all trees

#5 (Liver Transplantation)**:ti,ab,kw** OR (Hepatic Transplantation)**:ti,ab,kw** OR (Hepatic Transplantations)**:ti,ab,kw** OR (Liver Transplantations)**:ti,ab,kw** OR (Liver Transplant )**:ti,ab,kw** OR (Liver Transplants)**:ti,ab,kw** OR (Grafting Liver)**:ti,ab,kw**

#6 #4 OR #5

#7 #3 AND #6

#8 **MeSH descriptor: [Immune Checkpoint Inhibitors] explode all trees**

#9 (Immune Checkpoint Inhibitors):ti,ab,kw OR (**Immune Checkpoint Inhibitors)**:ti,ab,kw OR (Immune Checkpoint Blockers):ti,ab,kw OR (Immune Checkpoint Inhibitor):ti,ab,kw OR (CTLA-4 Inhibitors):ti,ab,kw OR (CTLA 4 Inhibitors):ti,ab,kw OR (Cytotoxic T-Lymphocyte-Associated Protein 4 Inhibitors):ti,ab,kw OR (Cytotoxic T Lymphocyte Associated Protein 4 Inhibitors):ti,ab,kw OR (Cytotoxic T-Lymphocyte-Associated Protein 4 Inhibitor):ti,ab,kw OR (Cytotoxic T Lymphocyte Associated Protein 4 Inhibitor):ti,ab,kw OR (CTLA-4 Inhibitor):ti,ab,kw OR (CTLA 4 Inhibitor):ti,ab,kw OR (PD-1 Inhibitors):ti,ab,kw OR (PD 1 Inhibitors):ti,ab,kw OR (Programmed Cell Death Protein 1 Inhibitor):ti,ab,kw OR (Programmed Cell Death Protein 1 Inhibitors):ti,ab,kw OR (PD-1 Inhibitor):ti,ab,kw OR (PD 1 Inhibitor):ti,ab,kw OR (Immune Checkpoint Blockade):ti,ab,kw OR (Immune Checkpoint Inhibition):ti,ab,kw OR (PD-L1 Inhibitors):ti,ab,kw OR (PD L1 Inhibitors):ti,ab,kw OR (Programmed Death-Ligand 1 Inhibitors):ti,ab,kw OR (Programmed Death Ligand 1 Inhibitors):ti,ab,kw OR (PD-L1 Inhibitor):ti,ab,kw OR (PD L1 Inhibitor):ti,ab,kw OR (PD-1PD-L1 Blockade):ti,ab,kw OR (PD 1 PD L1 Blockade):ti,ab,kw

#10 #8 OR #9

#11 **(atezolizumab)**:ti,ab,kw **OR (anti-PDL1)**:ti,ab,kw **OR (MPDL3280A)**:ti,ab,kw **OR (MPDL-3280A)**:ti,ab,kw **OR (Tecentriq)**:ti,ab,kw **OR (RG7446)**:ti,ab,kw **OR (RG-7446)**:ti,ab,kw

#12 MeSH descriptor: [Nivolumab] explode all trees

#13 (Nivolumab):ti,ab,kw **OR (MDX-1106)**:ti,ab,kw **OR (MDX1106)**:ti,ab,kw **OR (MDX 1106)**:ti,ab,kw **OR (Opdivo)**:ti,ab,kw **OR (BMS-936558)**:ti,ab,kw **OR (BMS936558)**:ti,ab,kw **OR (BMS 936558)**:ti,ab,kw **OR (ONO-4538)**:ti,ab,kw **OR (ONO4538)**:ti,ab,kw **OR (ONO 4538)**:ti,ab,kw

#14 #12 OR #13

#15 **(pembrolizumab)**:ti,ab,kw **OR (MK-3475)**:ti,ab,kw **OR (Keytruda)**:ti,ab,kw **OR (lambrolizumab)**:ti,ab,kw **OR (SCH-900475)**:ti,ab,kw

#16 MeSH descriptor: [Ipilimumab] explode all trees

#17 (Ipilimumab):ti,ab,kw OR (Anti-CTLA-4 MAb Ipilimumab):ti,ab,kw OR (Anti CTLA 4 MAb Ipilimumab):ti,ab,kw OR (Ipilimumab, Anti-CTLA-4 MAb):ti,ab,kw OR (MDX 010):ti,ab,kw OR (MDX-010):ti,ab,kw OR (MDX010):ti,ab,kw OR (MDX-CTLA-4):ti,ab,kw OR (MDX CTLA 4):ti,ab,kw OR (Yervoy):ti,ab,kw

#18 #16 OR #17

#19 **(sintilimab)**:ti,ab,kw **OR (IBI 308)**:ti,ab,kw **OR (IBI308)**:ti,ab,kw

#20**(camrelizumab)**:ti,ab,kw **OR (SHR-1210)**:ti,ab,kw **OR (SHR 1210)**:ti,ab,kw **OR (carrelizumab)**:ti,ab,kw

#21 **(durvalumab)**:ti,ab,kw **OR (MEDI4736)**:ti,ab,kw **OR (MEDI-4736)**:ti,ab,kw **OR (Imfinzi)**:ti,ab,kw

#**22 (tislelizumab)**:ti,ab,kw **OR (BGB-A317)**:ti,ab,kw

#23 #10 OR #11 OR #14 OR #15 OR #18 OR #19 OR #20 OR #21 OR #22

#24 #7 AND #23
