# Supplementary material for: Navigating the risks: a systematic review of immune checkpoint inhibitor therapy before liver transplant for hepatocellular carcinoma and its impact on allograft rejection and survival outcomes
Source: Front Oncol. 2025 Oct 29;15:1689820. doi: 10.3389/fonc.2025.1689820 (PMC12605506; doi:10.3389/fonc.2025.1689820)
Supplement: Supplementary file 2 [file DataSheet2.docx]

G


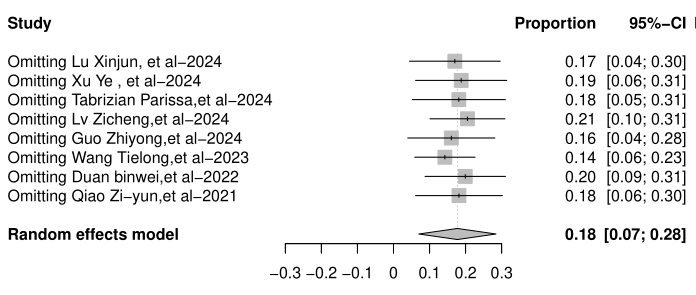

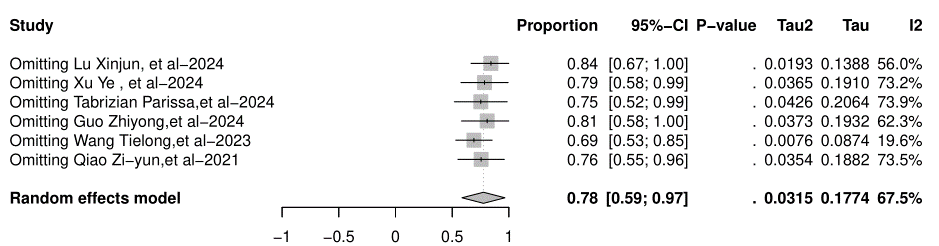

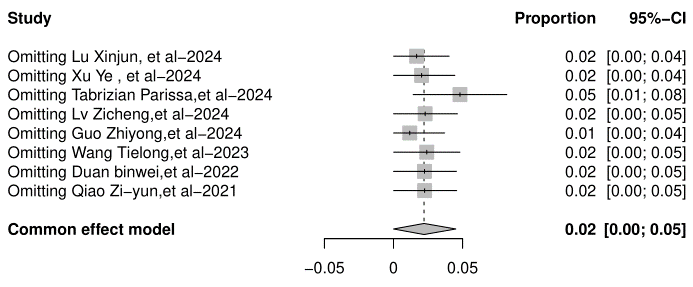


F


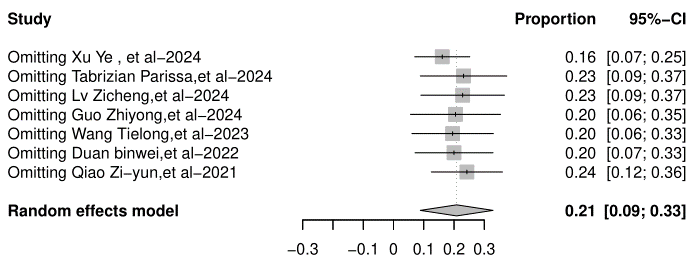

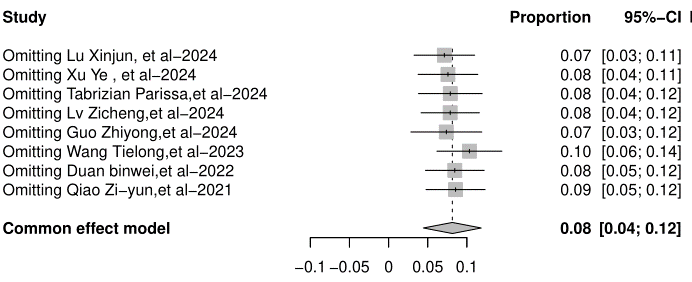


D


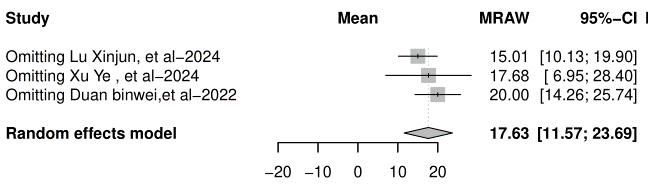

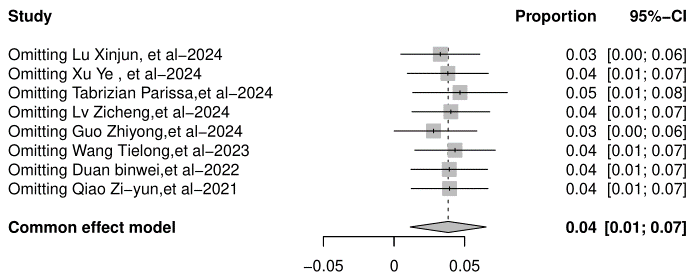


C

B

A

E

Supplementary Figure S1. Sensitivity analysis. (A) Sensitivity analysis for allograft rejection rate; (B) Sensitivity analysis for the full recovery rate; (C) Sensitivity analysis for graft loss;(D)Sensitivity analysis for HCC recurrence rate; (E) Sensitivity analysis for RFS;(F) Sensitivity analysis for mortality rate;(G) Sensitivity analysis for AR-related mortality.

Supplementary Figure S2. Funnel plots of publication bias. (A): Funnel plot of publication bias of allograft rejection rate; (B) Funnel plot of publication bias of the full recovery rate; (C) Funnel plot of publication bias of graft loss;(D) Funnel plot of publication bias of HCC recurrence rate; (E) Funnel plot of publication bias of RFS;(F) Funnel plot of publication bias of mortality rate;(G) Funnel plot of publication bias of AR-related mortality.

G

F

E

D

C

B

A


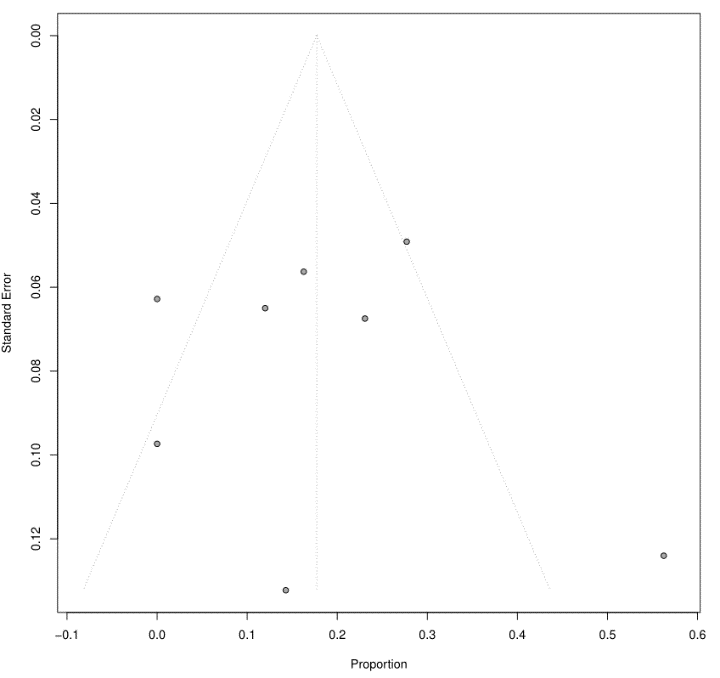

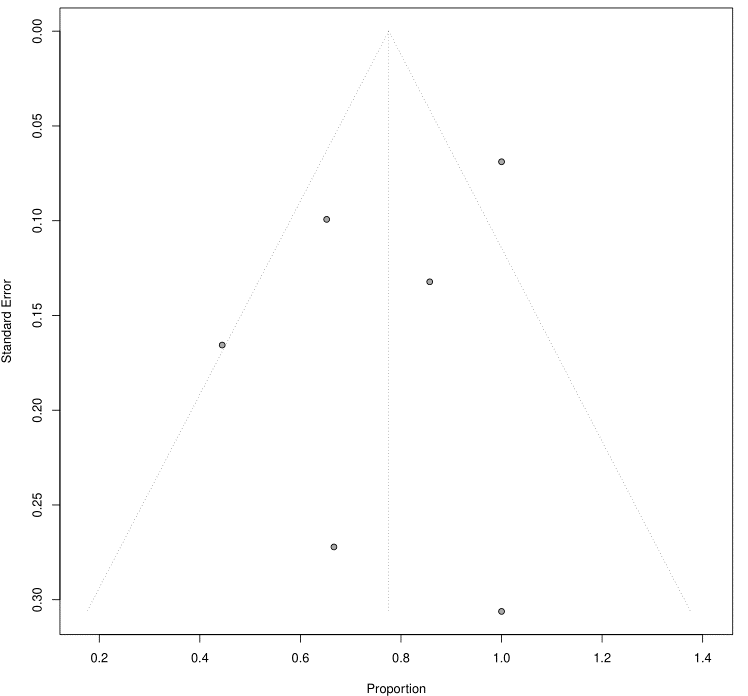

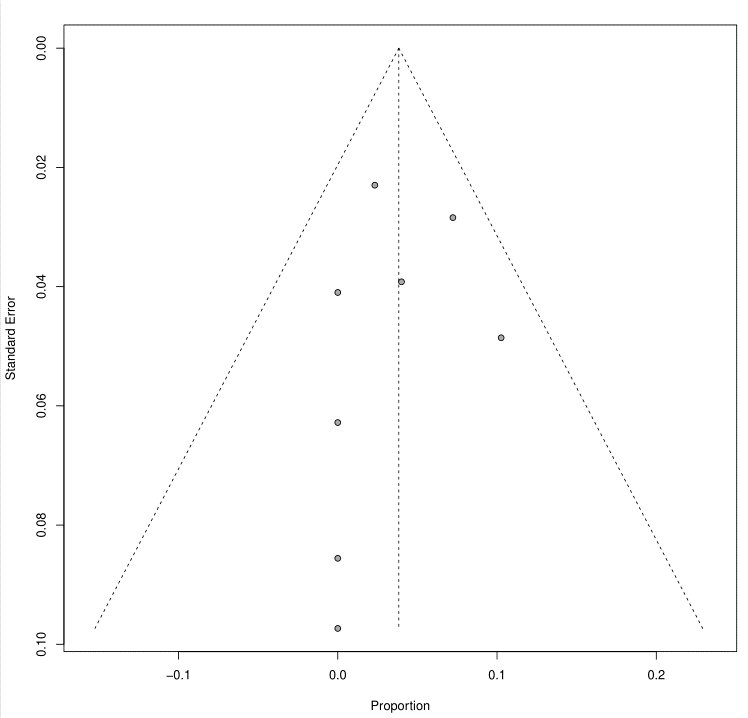

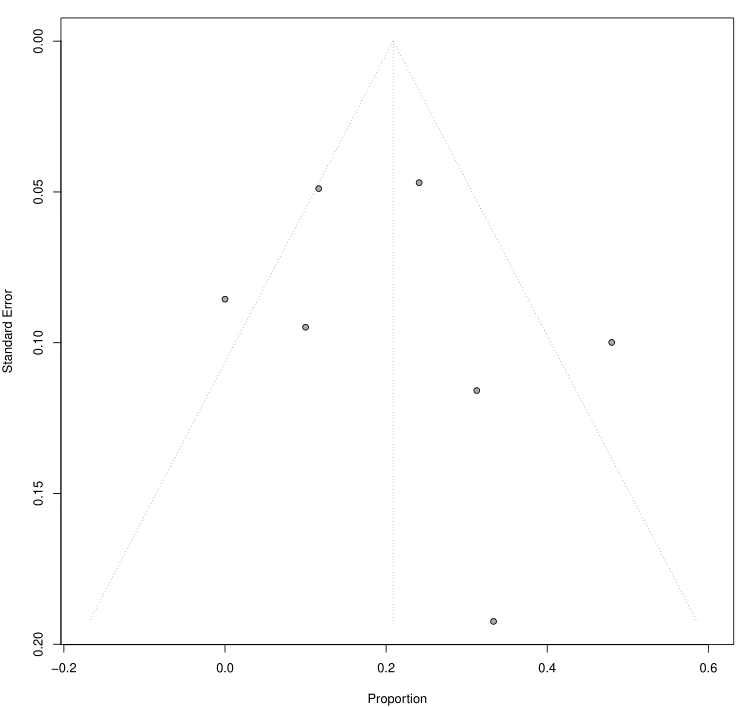

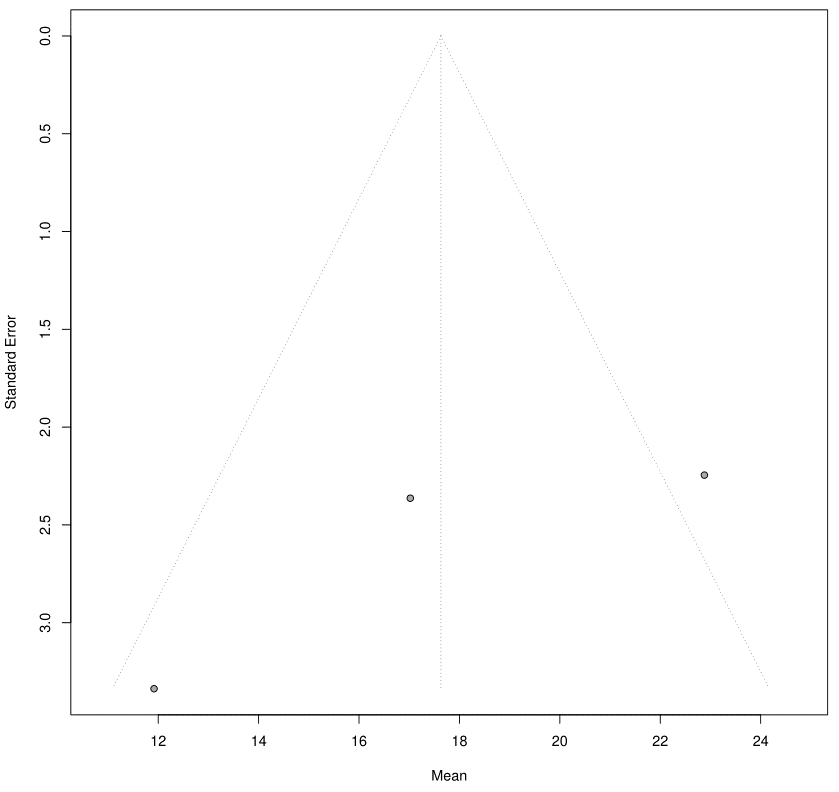

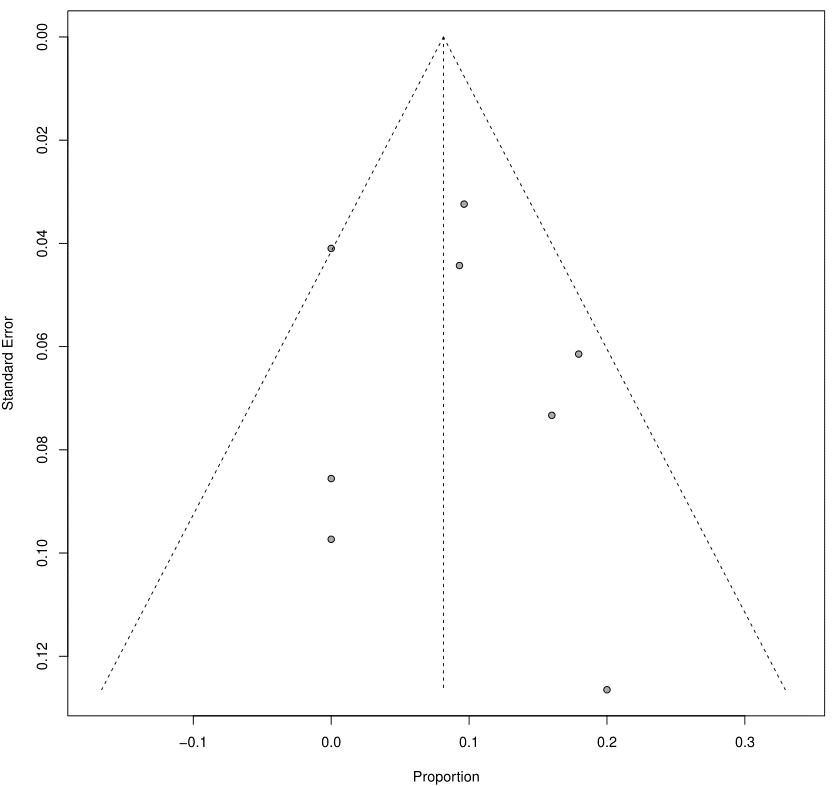

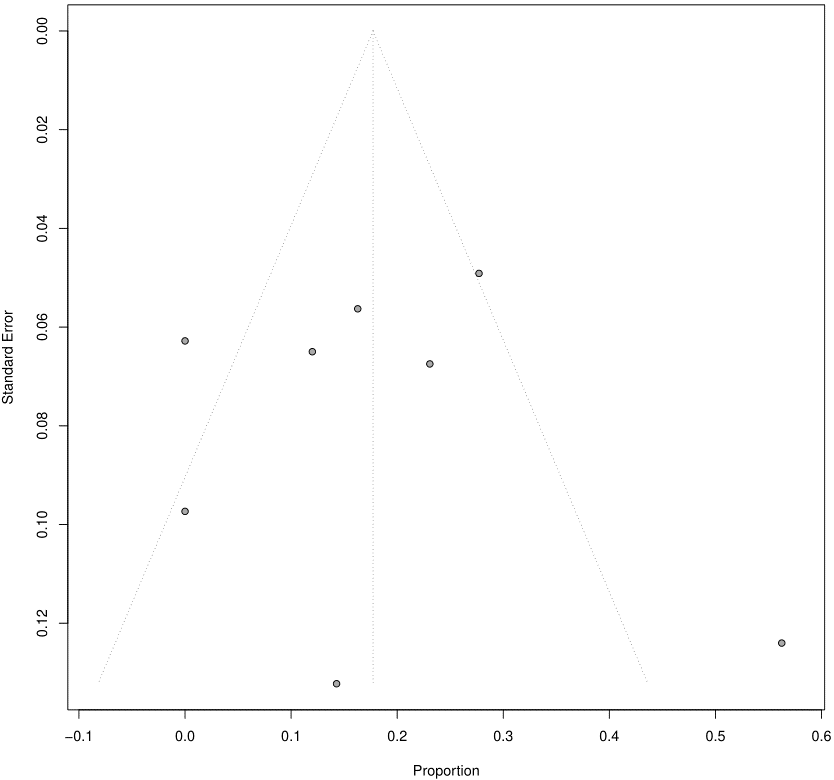


Supplementary Table S1. Treatment and prognosis of 52 recipients of rejection.

| Recipient ID | ICIs | Reason | IWP | LRTs | TKIs or  bevacizumab | Liver  Biopsy | RAI | Grade | POD  (d) | Outcome |
| --- | --- | --- | --- | --- | --- | --- | --- | --- | --- | --- |
| A01**^[14]^** | Camrelizumab | Downstaging | 87 | Yes | Yes | Yes | 4 | Mild | 21 | Death  (graft loss) |
| A02**^[14]^** | Sintilimab | Bridging | 19 | Yes | Yes | Yes | 4 | Mild | 4 | Complete recovery^c^ |
| A03**^[14]^** | Camrelizumab | Downstaging | 26 | Yes | Yes | Yes | 6 | Moderate | 35 | Death (graft  Dysfunction) |
| A04**^[14]^** | Atezolizumab | Downstaging | 62 | Yes | Yes | Yes | 8 | Severe | 143 | Death (graft  Dysfunction) |
| A05**^[[14]^** | Pembrolizumab | Downstaging | 17 | Yes | Yes | Yes | 4 | Mild | 8 | Complete recovery |
| A06**^[[14]^** | Pembrolizumab | Downstaging | 29 | Yes | Yes | Yes | 4 | Mild | 33 | Complete recovery |
| A07**^[14]^** | Pembrolizumab | Downstaging | 24 | - | Yes | Yes | 4 | Mild | 22 | Complete recovery |
| A08**^[[14]^** | Atezolizumab | Downstaging | 57 | Yes | Yes | No | / | / | 48 | Complete recovery |
| A09**^[14]^** | Camrelizumab | Downstaging | 54 | Yes | Yes | No | / | / | 23 | Complete recovery |
| A10**^[14]^** | Pembrolizumab | Downstaging | 71 | - | Yes | No | / | / | 10 | Complete recovery |
| A11**^[14]^** | Sintilimab | Downstaging | 7 | Yes | Yes | No | / | / | 11 | Death(graft Dysfunction) |
| A12**^[14]^** | Camrelizumab | Bridging | 11 | Yes | Yes | No | / | / | 55 | Death(graft Dysfunction) |
| A13**^[14]^** | Tislelizumab | Downstaging | 11 | Yes | Yes | No | / | / | 25 | Complete recovery |
| A14**^[14]^** | Camrelizumab | Downstaging | 126 | Yes | Yes | No | / | / | 17 | Complete recovery |
| A15**^[14]^** | Tislelizumab | Downstaging | 13 | Yes | Yes | No | / | / | 150 | Death(graft Dysfunction) |
| A16**^[14]^** | Pembrolizumab | Downstaging | 5 | Yes | Yes | No | / | / | 7 | Complete recovery |
| A17**^[14]^** | Nivolumab | Bridging | 18 | Yes | Yes | No | / | / | 5 | Complete recovery |
| A18**^[14]^** | Pembrolizumab | Downstaging | 21 | - | Yes | No | / | / | 9 | Complete recovery |
| A19**^[14]^** | Camrelizumab | Downstaging | 47 | Yes | Yes | No | / | / | 7 | Complete recovery |
| A20**^[14]^** | Pembrolizumab | Downstaging | 24 | Yes | Yes | No | / | / | 7 | Partial remission |
| A21**^[14]^** | Pembrolizumab | Bridging | 29 | - | Yes | No | / | / | 13 | Complete recovery |
| A22**^[14]^** | Sintilimab | Downstaging | 9 | Yes | Yes | No | / | / | 4 | Complete recovery |
| A23**^[14]^** | Sintilimab | Downstaging | 6 | Yes | Yes | No | / | / | 7 | Partial remission |
| A24**^[19]^** | Sintilimab | Downstaging | 7 | Yes | Yes | No | / | / | 10 | Death  (graft loss) |
| A25**^[19]^** | Pembrolizumab | Downstaging | 6 | Yes | Yes | No | / | / | 9 | Complete recovery |
| A26**^[19]^** | Pembrolizumab | Downstaging | 3 | Yes | Yes | Yes | 6 | / | 14 | Complete recovery |
| A27**^[19]^** | Toripalimab | Downstaging | 14 | NO | Yes | No | / | / | 10 | Death  (graft loss) |
| A28**^[19]^** | Sintilimab | Downstaging | 80 | Yes | Yes | Yes | 6 | / | 14 | Complete recovery |
| A29**^[19]^** | Camrelizumab, Tislelizumab | Downstaging | 7 | Yes | Yes | No | / | / | 16 | Death (Secondary infection) |
| A30**^[19]^** | Tislelizumab | Downstaging | 15 | Yes | Yes | Yes | 7 | / | 8 | Death  (graft loss) |
| A31**^[19]^** | Camrelizumab | Downstaging | 65 | Yes | Yes | Yes | 4 | / | 7 | Complete recovery |
| A32**^[19]^** | Sintilimab | Downstaging | 74 | Yes | Yes | Yes | 9 | / | 8 | Death  (graft loss) |
| A33**^[13]^** | Camrelizumab  Nivolumab | Downstaging | 14 | Yes | Yes | Yes | 5 | / | 11 | Death  (graft loss) |
| A34**^[13]^** | Atezolizumab  Pembrolizumab | Downstaging | 37 | Yes | Yes | Yes | 4 | / | 48 | Complete recovery |
| A35**^[13]^** | Pembrolizumab | Downstaging | 43 | Yes | Yes | Yes | 5 | / | 124 | Death  (recurrence) |
| A36**^[15]^** | Nivolumab | / | 43 (13-120) | Yes | / | Yes | / | Grade 2:2  Grade 3:3  Grade 4:2 | 4 (7-132 ) | 6 complete recovery and one required retransplantation |
| A37**^[15]^** | Nivolumab | / |  | Yes | / | Yes | / |  |  |  |
| A38**^[15]^** | Nivolumab | / |  | Yes | / | Yes | / |  |  |  |
| A39**^[15]^** | Nivolumab | / |  | Yes | / | Yes | / |  |  |  |
| A40**^[15]^** | Nivolumab | / |  | Yes | / | Yes | / |  |  |  |
| A41**^[15]^** | Atezolizumab/  Bevacizumab | / |  | Yes | / | Yes | / |  |  |  |
| A42**^[15]^** | Atezolizumab/  Bevacizumab | / |  | Yes | / | Yes | / |  |  |  |
| A43**^[20]^** | Pembrolizumab | Downstaging | 24 | Yes | Yes | Yes | 4 | / | 22 | Complete recovery |
| A44**^[20]^** | Pembrolizumab | Downstaging | 29 | Yes | Yes | No | / | / | 15 | Complete recovery |
| A45**^[20]^** | Camrelizumab | Downstaging | 90 | Yes | Yes | Yes | 6 | / | 7 | Complete recovery |
| A46**^[20]^** | Sintilimab | Downstaging | 26 | Yes | Yes | Yes | 6 | / | 4 | Complete recovery |
| A47**^[20]^** | Sintilimab | Downstaging | 14 | Yes | Yes | No | / | / | 4 | Complete recovery |
| A48**^[20]^** | Sintilimab | Downstaging | 21 | Yes | Yes | No | / | / | 9 | Complete recovery |
| A49**^[20]^** | Pembrolizumab | Downstaging | 20 | Yes | Yes | No | / | / | 4 | Complete recovery |
| A50**^[20]^** | Pembrolizumab | Downstaging | 7 | Yes | NO | No | / | / | 7 | Complete recovery |
| A51**^[20]^** | Pembrolizumab | Downstaging | 17 | Yes | NO | Yes | 4 | / | 8 | Complete recovery |
| A52**^[22]^** | Camrelizumab  or pembrolizumab | Downstaging | / | / | Yes | Yes | 5 | Mild | 11 | Complete recovery |

Abbreviation: ICIs, immune checkpoint inhibitors; IWP, ICIs washout period; TKIs, tyrosine kinase inhibitors; LRTs, locoregional therapies; POD, postoperative days;

Complete recovery: after undergoing antirejection treatment, the symptoms during the rejection period, such as nausea, fatigue, jaundice, etc., disappear, and the levels of alanine aminotransferase/serum aspartate aminotransferase and total bilirubin gradually return to baseline or near-baseline level after the rejection has been controlled, and this is maintained for 1 week or longer

Supplementary Table S2. Case series of ICIs before liver transplantation.

| Study (Year published) | Country | Number of Patients (Rejections) | Age/Sex | ICIs | ICIs cycles | Washout Period | Rejection proved by biopsy | Retransplantation |
| --- | --- | --- | --- | --- | --- | --- | --- | --- |
| Kulkarni et al^[41]^  (2024) | India | 5(0) | 57  (range:41-62); 4M/1F | Atezolizumab-Bevacizumab | 5 (range :3-7) | 79  (range:38-114) d | - | - |
| Tabrizian et al^[42]^(2021) | USA | 9(1) | NR | Nivolumab | NR | 22 | NR | NR |
| Schnickel^[43]^ (2022) | USA | 5 (2) | 60/F  65/M | Nivolumab | NR | 35  10 | POD 14  <POD 14 | No,  corticosteroids  No, ATG,  rituximab, or  IVIG |
| Dave et al.^[44]^  (2022) | USA | 5 (2) | Mean age 61  ±6.52;  NR | Nivolumab | NR  NR | <90  <90 | Yes  Yes | Yes, successful  No, death |
| Chen et al^[45]^  (2021) | China | 5 (0) | Mean age  53.2 ±5.4;4M/ 1F | Nivolumab | NR | 63.80 ± 18.3 | - | - |

Figures are number (percentage), median (interquartile range or range) or mean±standard.

Abbreviation: NR, not reported; ATG, antithymocyte globulin; IVIG, intravenous Immunoglobulin; POD, postoperative days
